# Optimal allocation of clusters in stepped wedge designs with a decaying correlation structure

**DOI:** 10.1371/journal.pone.0289275

**Published:** 2023-08-16

**Authors:** Mirjam Moerbeek

**Affiliations:** Department of Methodology and Statistics, Utrecht University, Utrecht, The Netherlands; ICAR-CNR: Istituto di Calcolo e Reti ad Alte Prestazioni Consiglio Nazionale delle Ricerche, ITALY

## Abstract

The cluster randomized stepped wedge design is a multi-period uni-directional switch design in which all clusters start in the control condition and at the beginning of each new period a random sample of clusters crosses over to the intervention condition. Such designs often use uniform allocation, with an equal number of clusters at each treatment switch. However, the uniform allocation is not necessarily the most efficient. This study derives the optimal allocation of clusters to treatment sequences in the cluster randomized stepped wedge design, for both cohort and cross-sectional designs. The correlation structure is exponential decay, meaning the correlation decreases with the time lag between two measurements. The optimal allocation is shown to depend on the intraclass correlation coefficient, the number of subjects per cluster-period and the cluster and (in the case of a cohort design) individual autocorrelation coefficients. For small to medium values of these autocorrelations those sequences that have their treatment switch earlier or later in the study are allocated a larger proportion of clusters than those clusters that have their treatment switch halfway the study. When the autocorrelation coefficients increase, the clusters become more equally distributed across the treatment sequences. For the cohort design, the optimal allocation is almost equal to the uniform allocation when both autocorrelations approach the value 1. For almost all scenarios that were studied, the efficiency of the uniform allocation is 0.8 or higher. R code to derive the optimal allocation is available online.

## Introduction

The cluster randomized trial [1–5] is often used in clinical, health and social science. It randomizes clusters, such as schools, family practices or households, rather than individuals to experimental conditions. This design is often chosen for political, ethical, financial reasons [6] and to avoid or reduce the risk of contamination of the control condition [7, 8]. Randomization of clusters comes at a price, though, namely a reduced efficiency due to dependencies among outcome measurements of subjects within the same cluster.

One approach to compensate for the loss of efficiency is the implementation of a multi-period design, such as a cross-over design [9, 10] or a stepped wedge design [11]. The stepped wedge design is a uni-directional switch design in which all clusters start in the control condition. Clusters are often randomized to the treatment sequences in advance and at the beginning of each new time period a number of clusters crosses over to the intervention condition. At the end of the trial all clusters receive the intervention condition. As logistic constraints often prevent the simultaneous enrollment of the intervention in all clusters, crossing over to the intervention is done in steps. This design is often used in pragmatic trials in health services research when the intervention is strongly believed to be effective and it is considered to be unethical to refrain some clusters from receiving it.

**Fig 1:**
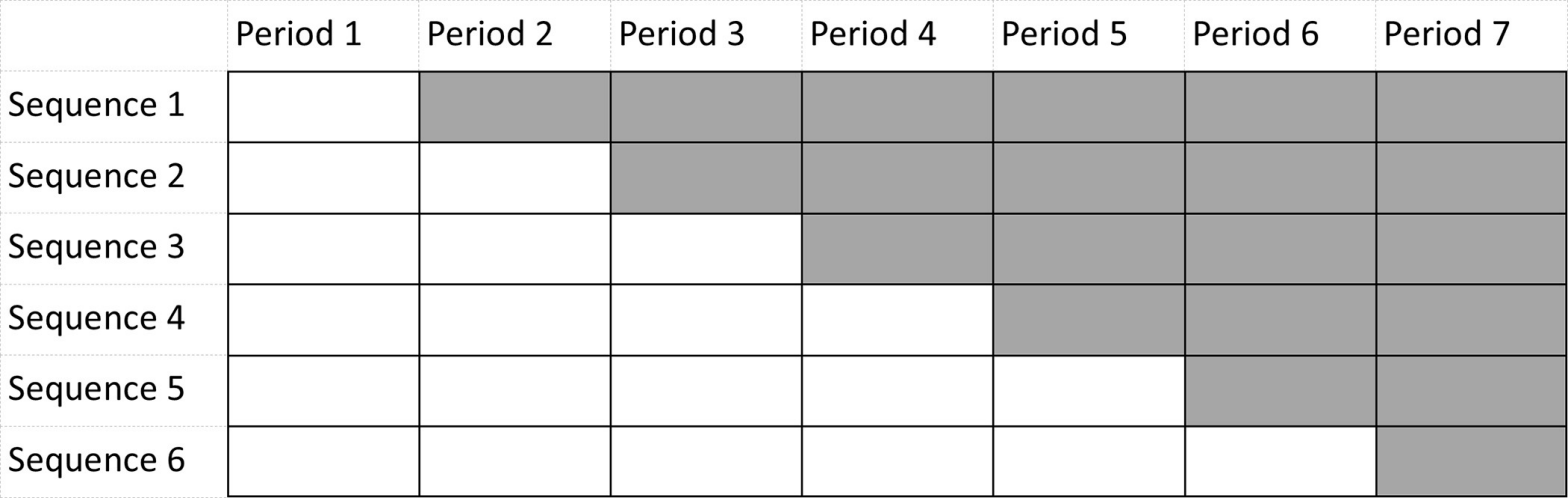
Graphical representation of a stepped-wedge design with 6 sequences.

A graphical representation of a stepped wedge design is given in Fig 1. There are seven time periods and six treatment sequences. In time period 1 all clusters are in the control condition (white cells). At the beginning of each subsequent time period a number of them crosses over to the intervention condition (grey cells). At the end of the trial all clusters are in the intervention condition. As both conditions are available within each treatment sequence, and hence within each cluster, the stepped wedge design is more efficient than the parallel-group design with the same number of time periods. Furthermore, as all clusters ultimately receive the promising intervention, recruitment may be easier than in the parallel-group design.

Stepped wedge designs are often designed to be uniform, meaning that the number of clusters per treatment switch is constant. A rationale for the uniform allocation is that it is consistent with the view of clinical equipoise that must exist before the start of the trial [12]. However, the uniform allocation is not necessarily an efficient choice, especially so when costs and/or variances differ across the conditions [13]. In the second half of the past decade a few papers focused on the optimal allocation of clusters to treatment sequences [14–17]. These papers assumed a rather simple correlation structure at the within-cluster and within-individual levels. Correlations between outcomes of two subjects in the same cluster but in distinct time periods did not depend on the time lag between these periods. Similarly, correlations between two measurements of the same individual but in distinct time periods did not depend on the time lag between these periods. Since the publication of these papers, a more realistic exponential decay correlation structure has been proposed. This correlation structure allows the correlation between two measurements to exponentially decrease as a function of the time lag between them [18, 19]. This is often seen in longitudinal trials where the dependency of two measurements becomes smaller if these are further apart in time.

When deriving the optimal allocation of clusters to treatment sequences, it is important the correlation structure is correctly specified. In other words, the optimal allocations as presented in papers [14–17] may be suboptimal when the correlation structure is exponential. The aim of this contribution is to provide tools to derive the optimal allocation of clusters to treatment sequences in cluster randomized stepped wedge designs given the exponential decay correlation structure. This will be done for cohort studies, where the same subjects are measured at the end of each time period, and for cross-sectional designs, where different sets of subjects are measured at the end of each time period. Furthermore, the efficiency of the uniform allocation is studied.

The structure of this contribution is as follows. In the next section the statistical model is presented along with the exponential decay function. The second half of this section focusses on optimal design methodology. The criterion to be minimized is the variance of the treatment effect estimator and it is explained how the optimal allocation is found. In the results section the optimal allocations are presented for the cross-sectional and cohort design as a function of the autocorrelation coefficients and for various realistic values of the intraclass coefficient and number of subjects per cluster-period. This section also shows how large the loss in efficiency is from using the uniform rather than the optimal allocation. The discussion section summarizes the results, discusses the limitation of the study and gives directions for future research.

## Methods

### Statistical model

The focus is on stepped wedge designs with *S* sequences. All clusters start in the control and a number of them switch over to the intervention at each time period, hence the number of time periods is *T* = *S*+1. Implementation periods are not included in the trial. Measurements are taken at the end of each time period.

The data have a nested structure, and are analyzed using a multilevel model [20–22], which is also known as a hierarchical linear model [23]. This model assumes an sufficient amount of clusters so that the clusters can be represented by random effects. For small number of clusters (say less than 20) it is suggested to represent the clusters by fixed rather than random effects [21]. The statistical model for individual *i* = 1,…,*m* in period *t* = 1,…,*T* in cluster *k* = 1,…,*K* is

$$Y_{kti}=\beta_{t}+\theta X_{kt}+{CP}_{kt}+\varepsilon_{kti},$$

where *β*_*t*_ is the fixed effect of period *t*, *X*_*kt*_ is treatment indicator (with values 0 and 1 for the control and intervention condition, respectively), *θ* is the fixed treatment effect, ${CP}_{kt}∼N(0,\sigma_{CP}^{2})$ is the cluster-period random effect. These effects are assumed independent if they come from different clusters *k* and $k^{\prime }:cov({CP}_{kt},{CP}_{k^{\prime }t})=0$. Furthermore, $\varepsilon_{kti}∼N(0,\sigma_{\varepsilon }^{2})$ is the individual-level error. Individual-level errors from different individuals *i* and *i*′ are assumed independent: *cov*(*ε*_*kti*_, *ε*_*kti′*_) = 0.

The discrete-time exponential decay function [19] is used to model the within-cluster and, in the case of a cohort design, the within-individual correlation structure. The covariance between two different subjects in the same cluster is $cov({CP}_{kti},{CP}_{kt\prime i\prime })=\sigma_{CP}^{2}\pi^{\left|t-t\prime \right|}$, with *π*∈[0, 1]. For *t* = *t*′ it reduces to $\sigma_{CP}^{2}$, which is the covariance of two subjects within the same cluster-period. The intraclass correlation coefficient is calculated by dividing this covariance by the total amount of variance: $\rho =\sigma_{CP}^{2}/(\sigma_{CP}^{2}+\sigma_{\varepsilon }^{2})$. It quantifies the proportion variance at the cluster level. It is a measure of dependency among subjects in the same cluster and period. The covariance between two individual-level errors from the same subject in a cohort design is $cov(\varepsilon_{kti},\varepsilon_{kt\prime i})=\sigma_{\varepsilon }^{2}\tau^{\left|t-t\prime \right|}$, with *τ*∈[0,1]. With the cross-sectional model each individual is measured only once, so *cov*(*ε*_*kti*_, *ε*_*kt′i*_) is not defined. The parameters *π* and *τ* are called the cluster and individual autocorrelations, respectively. They are assumed constant across clusters and individuals, hence they do not have subscripts *i* and *k*. When autocorrelations are equal to 1, the correlation between any two measurements does not depend on the time lag between these measurements. This is called the exchangeable correlation structure.

The model can also be presented in terms of cluster-period means [19, 24].

$${\bar{Y}}_{kt.}=\beta_{t}+\theta X_{kt}+{CP}_{kt}+{\bar{\varepsilon }}_{kt.},$$

where

$${\bar{Y}}_{kt.}=\frac{1}{m}\sum_{i=1}^{m}Y_{kti}$$

is the mean in cluster-period *t* in cluster *k*, *β*_*t*_ is the fixed effect of period *t*, *X*_*kt*_ is treatment indicator, *θ* is the fixed treatment effect, *CP*_*kt*_ is the cluster-period random effect and

$${\bar{\varepsilon }}_{kt.}=\frac{1}{m}\sum_{i=1}^{m}\varepsilon_{kti}$$

is the mean individual-level error in cluster-period *t* in cluster *k*. The latter has variance

$$var({\bar{\varepsilon }}_{kt.})=\frac{\sigma_{\varepsilon }^{2}}{m},$$

and the covariance of two measurements in distinct time periods is

$$cov({\bar{\varepsilon }}_{kt.},{\bar{\varepsilon }}_{kt\prime .})=\frac{\sigma_{\varepsilon }^{2}\tau^{\left|t-t\prime \right|}}{m}.$$

The *T*×*T* covariance matrix of responses in cluster *k* is denoted *V* and for the cohort design it is defined as

$$V=\frac{\sigma_{\varepsilon }^{2}}{m}R(\tau )+\sigma_{CP}^{2}R(\pi ),$$

with

$$R(\tau )=\left(\begin{array}{ccccc} 1 & \tau & \tau^{2} & \vdots & \tau^{T-1}\\ \tau & 1 & \tau & \tau^{2} & \vdots \\ \tau^{2} & \tau & 1 & \tau & \tau^{2}\\ \vdots & \tau^{2} & \tau & 1 & \tau \\ \tau^{T-1} & \vdots & \tau^{2} & \tau & 1 \end{array}\right)$$

and

$$R(\pi )=\left(\begin{array}{ccccc} 1 & \pi & \pi^{2} & \vdots & \pi^{T-1}\\ \pi & 1 & \pi & \pi^{2} & \vdots \\ \pi^{2} & \pi & 1 & \pi & \pi^{2}\\ \vdots & \pi^{2} & \pi & 1 & \pi \\ \pi^{T-1} & \vdots & \pi^{2} & \pi & 1 \end{array}\right).$$

For the cross-sectional design the matrix *R*(*τ*) is replaced by the identity matrix *I* of dimension *T*×*T*. Note that in the extreme case where both autocorrelations *τ* and *π* are equal to 1, all elements in *R*(*τ*) become equal to $\tau^{\left|t-t\prime \right|}=1^{\left|t-t\prime \right|}=1$ and similarly all elements in *R*(*π*) become equal to $\pi^{\left|t-t\prime \right|}=1^{\left|t-t\prime \right|}=1$. The covariance matrix of the responses then becomes proportional to a matrix where every entry is equal to 1. Such a matrix cannot be inverted, meaning the model cannot be fitted to the data.

The variance of the estimator of the fixed effects *γ* = (*β*_1_, *β*_2_,…,*β*_*T*_, *θ*)′ is [25]

$$var(\hat{\gamma })={\left(\sum_{s=1}^{S}Np_{s}X_{s}\prime V^{-1}X_{s}\right)}^{-1}$$

where = (*X*_*k*1_, *X*_*k*2_,…,*X*_*kT*_)′ is the vector of treatment assignment of cluster *k* in sequence *s* = 1,…,*S* = *T*−1 and *p*_*s*_ is the proportion clusters in sequence *s*.

### Optimal design methodology

#### Optimality criterion

Optimal design methodology is used to derive the optimal allocation of clusters to treatment sequences [26, 27]. As the primary interest is in the effect of treatment, *θ*, the variance of its estimator, $var(\hat{\theta })$, is used a optimality criterion. The aim is to minimize this variance, as smaller variance results in higher efficiency of the design and hence larger power to detect an effect of treatment. This variance appears in the lower right cell of the matrix $var(\hat{\gamma })$ and depends on the intraclass correlation coefficient *ρ*, the number of subjects *m* per cluster-period and the two autocorrelation coefficients *τ* and *π*.

The proportion of clusters in treatment sequence *S* is denoted *p*_*s*_. An allocation of clusters to treatment sequences is denoted *p*, with *p* = (*p*_1_, *p*_2_,…,*p*_*S*_), for which the inequality constraint 0≤*p*_*s*_≤1, ∀*s* and equality constraint $\sum_{s=1}^{S}p_{s}=1$ hold. The optimal allocation is denoted $p^{*}=(p_{1}^{*},p_{2}^{*},\ldots ,p_{S}^{*})\prime$ and it is the one among all possible allocations *p* = (*p*_1_, *p*_2_,…,*p*_*S*_) for which $var(\hat{\theta })$ is minimized.

A simple mathematical expression for the optimal allocation p* cannot be derived, hence the optimal allocation has to be found numerically. R script that uses the function constrOptim.nl for minimizing nonlinear functions with equality and inequality constraints from the package alabama [28] can be found on my GitHub page https://github.com/MirjamMoerbeek/Optimal_CRSWD.

The efficiency of the uniform allocation versus the optimal allocation is calculated by dividing the value of $var(\hat{\theta })$ as obtained with the optimal allocation by the value of $var(\hat{\theta })$ as obtained with the uniform allocation. This gives a value between 0 and 1, where high values indicate the uniform allocation is highly efficient.

## Results

Once a choice has been made between a cohort and cross-sectional design, the number of sequences *S* and individuals *m* per cluster-period have been determined, and a priori estimates of the intraclass correlation *ρ*, and autocorrelations *π* and *τ* are available, the optimal allocation can be derived. Various papers present estimates of the intraclass correlation coefficient *ρ* in various fields of science and for various types of clusters; an overview is presented in Table 11.1 of [29]. These can help the researcher to get an a plausible priori estimate of *ρ*. Unfortunately, less guidance is available in the literature to obtain a priori estimates of the individual and cluster autocorrelations *π* and *τ*. For that reason, optimal allocations are presented as a function of these two model parameters within their intervals [0, 1]. The optimal allocation and efficiency of the uniform allocation versus the optimal allocation are studied for *S* = 3, 4, 5 and 6 sequences, *m* = 5,25 and 50 individuals per cluster-period, and intraclass correlation coefficient *ρ* = 0.0125, 0.025 and 0,05. This results in 3×3×4 = 36 scenarios. These scenarios were considered for both the cross-sectional design and cohort design. Extensive results are presented in the online supporting information S1 and S2 Appendices.

### Cross-sectional design

**Fig 2:**
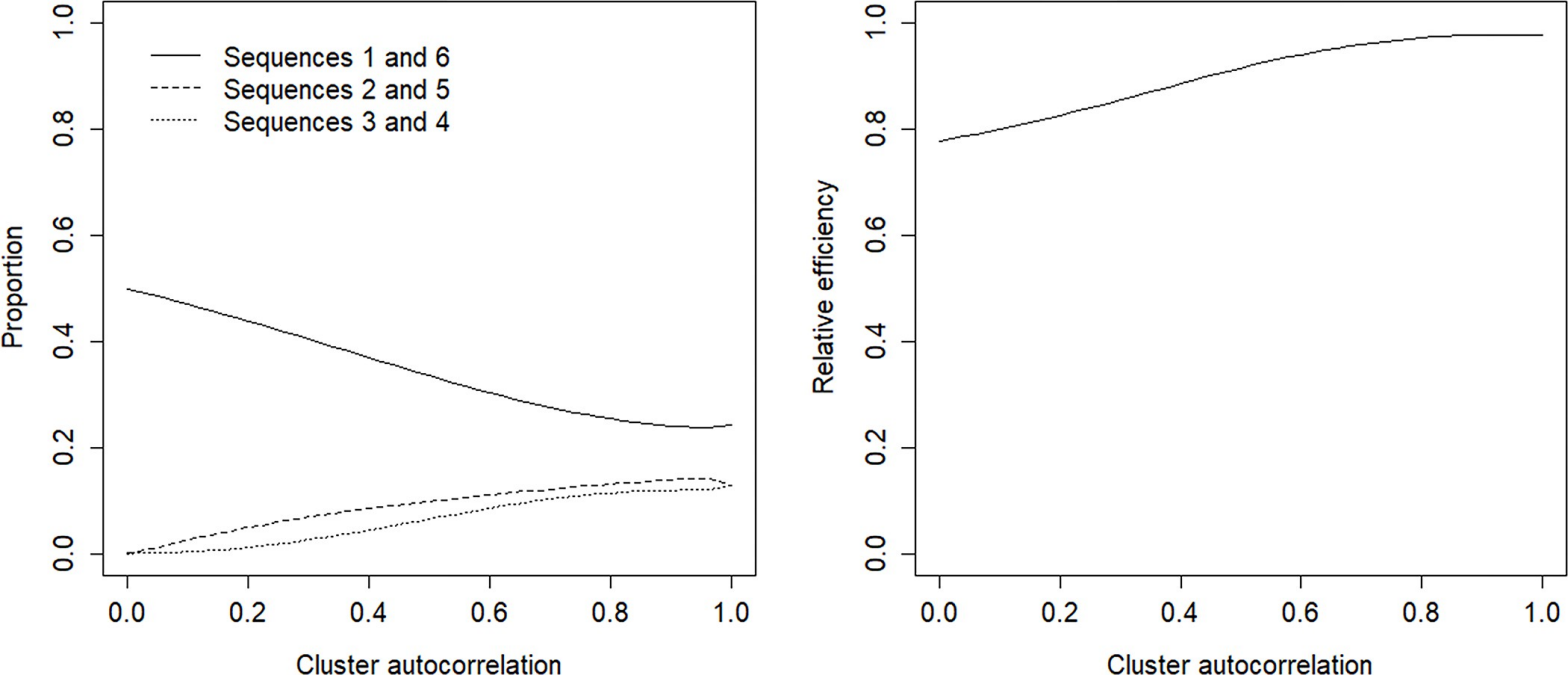
Optimal allocation of clusters and efficiency of uniform allocation in a cross-sectional design.

For the cross-sectional design the results for the 36 scenarios follow a general tendency, which is further discussed on the basis of the results for the scenario with *S* = 6 sequences, *m* = 25 individuals per cluster-period and intraclass correlation coefficient *ρ* = 0.05 as presented in Fig 2. The left panel shows the optimal allocation, the right panel the efficiency of the uniform allocation.

For any value of the cluster autocorrelation, most clusters are assigned to sequences 1 and 6, followed by sequences 2 and 5, and finally sequences 3 and 4. In other words, the optimal allocation is symmetric and more clusters are assigned to those sequences that have their treatment switch earlier or later in the study than to those that have their treatment switch halfway [30–32]. The optimal allocation depends on the size of the cluster autocorrelation. In other words, the optimal allocation for the exchangeable correlation structure (i.e. *π* = 1) does not hold for the exponential correlation structure (i.e. *π*<1). For low cluster autocorrelation almost all clusters are assigned to sequences 1 and 6, but if the cluster autocorrelation increases, the clusters become more equally distributed across the sequences. This is especially so for large *m* and/or large *ρ*, see the extensive results in the online supporting information S1 Appendix. However, for none of the 36 scenarios that were studied, the optimal allocation for large cluster autocorrelation approaches, or is equal to, the uniform allocation.

The right panel of Fig 2 shows the relative efficiency of the uniform allocation. It increases from about 0.8 for small cluster autocorrelation, to larger values when the cluster autocorrelation increases to 1. The uniform allocation has high efficiency when the cluster autocorrelation is large, but it may result in a loss of efficiency for small cluster autocorrelation. A relative efficiency of 0.8 implies 100%(1/0.8−1) = 25% extra clusters are needed to let the uniform allocation perform as well as the optimal allocation.

### Cohort design

In the cohort design individuals are measured multiple times, meaning the optimal allocation does not only depend on the within-cluster autocorrelation but also the within-individual autocorrelation. Optimal allocations are therefore presented by means of contour plots. Results for all 36 scenarios can be found in the online supporting information S1 Appendix.

**Fig 3:**
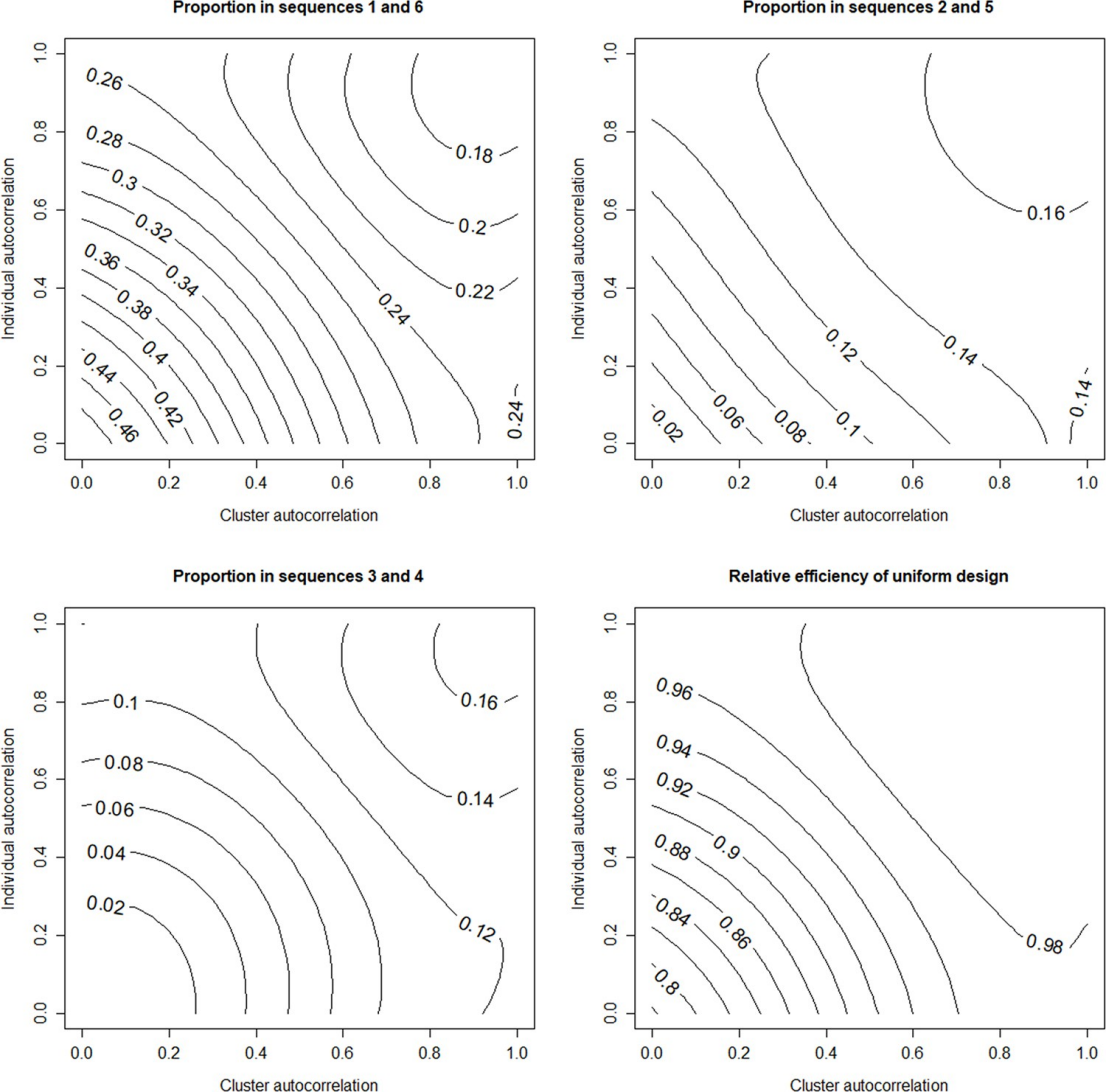
Optimal allocation of clusters and efficiency of uniform allocation in a cohort design.

Again, some general findings can be observed, which will be further discussed on the basis of the scenario with *S* = 6, *m* = 25 and *ρ* = 0.05 (see Fig 3). The two top panels and the one bottom left show optimal allocations, the one bottom right shows the efficiency of the uniform allocation. The optimal allocation is symmetric with equal proportion of clusters in sequences 1 and *S*, in sequences 2 and *S*−1, etcetera. Furthermore, sequences receive a higher proportion of clusters when they are nearer to the edges of the design. The optimal allocation depends on the two autocorrelation coefficients. When both autocorrelation coefficients are small, most clusters are allocated to sequences 1 and 6, and hardly any to the other four sequences. When one or both autocorrelations increase, the number of clusters allocated to sequences 1 and 6 becomes smaller, so more clusters are allocated to the other four sequences. When both autocorrelations approach the value 1 (meaning the correlation structure is exchangeable), the optimal allocation approaches the uniform allocation. In other words, the uniform allocation performs almost as well as the optimal allocation for large cluster and subject autocorrelation. However, when autocorrelations are small, the optimal allocation should be preferred. This is also illustrated by the relative efficiency, which is below 0.8 when both intraclass correlations are near 0, and increases to 1 when both intraclass correlations increase to 1.

### Illustrative example

The optimal allocations in the Results section were expressed in proportions *p*_*s*_, *s* = 1,…,*S* rather than the number of clusters per treatment sequence. This is a so-called continuous optimal design. The number of clusters *K*_*s*_ in each treatment sequence *s* = 1,…,*S* is found by calculating *K*_*s*_ = *Kp*_*s*_ and rounding off. In this section we use a proposed intensive care trial [19] as an example to illustrate how this can be done for a relatively large number of clusters. In this trial 33 intensive care units participated in a study with four periods of six months each and the outcome was length of stay. The exponential decay function was found to provide a reasonable approximation to the data. The design was a cross-sectional design. The intraclass correlation coefficient was estimated to be *ρ* = 0.035 and the cluster autocorrelation was estimated to be *π* = 0.95. When *m* = 500 patients per cluster-period are included, then the optimal allocation is $p^{*}={\left(p_{1}^{*},p_{2}^{*},p_{3}^{*},p_{4}^{*}\right)}^{\prime }=(0.409,0.091,0.091,0.409)\prime$. Multiplying by *K* = 33 clusters gives $K^{*}={\left(K_{1}^{*},K_{2}^{*},K_{3}^{*},K_{4}^{*}\right)}^{\prime }=(13.481\;3.019\;3.019\;13.481)\prime$. Each of these cluster sizes can be rounded upwards or downwards to the nearest integer. This gives a total of 2^4^ = 16 possible combinations of rounded cluster sizes, but only for four of these combinations the total number of clusters is *K* = 33. For each of these the variance $var(\hat{\theta })$ can be calculated. It turns out that the designs with K* = (13,3,4,13)′ and K* = (13,4,3,13)′ have the lowest variance and hence are the optimal designs. In these designs the number of clusters in one of the middle two sequences is rounded upwards, while the number of clusters in the other sequences are rounded downwards.

### Conclusions and discussion

The aim of this paper was to derive optimal allocations of clusters to treatment sequences in stepped wedge designs. The exponential decay correlation structure allows for smaller correlations when measurements are further apart in time. It may therefore be considered a plausible correlation structure in stepped wedge designs. Previous research on optimal allocation to sequences did not take into account this correlation structure.

Optimal allocations were presented for cross-sectional and cohort designs. For both types of design, treatment sequences that have their treatment switch earlier or later in time get assigned a higher number of clusters than those that have their treatment switch halfway the study. This is especially the case for small values of the autocorrelations. When autocorrelations increase, the clusters become more equally distributed over the treatment sequences. For the cohort design the allocation approaches the uniform allocation when both autocorrelations approach the value 1. For the cross-sectional design, however, the optimal allocation does no approach the uniform allocation for any of the scenarios studied. For some of the studied scenarios, the efficiency of the uniform allocation, relative to the optimal allocation, was somewhat below 0.8 at low values of the autocorrelation coefficients, meaning the uniform allocation may sometimes result in a considerable loss of efficiency. However, when autocorrelation coefficients are above 0.5, the relative efficiency was often above 0.9, meaning the loss in efficiency was small.

To use the methodology in this paper, a priori estimates of the intraclass correlation coefficient and autocorrelation coefficients must be specified. It is therefore important such estimates become available in the literature. I therefore encourage researchers to clearly report the estimates of the intraclass and autocorrelation coefficients to facilitate efficient planning of future studies. In the case of absence of such estimates, one can specify a range, rather than a single value, of plausible values of these model parameters and perform a sensitivity study to see to what degree the optimal allocation is affected by the model parameters. One can also approach the uncertainty in multiple model parameters by using a more formal and robust approach to derive the optimal allocation, such as a maximin optimal design [33].

The optimal allocations in the results section are expressed in proportions *p*_*s*_, *s* = 1,…,*S* rather than the number of clusters per treatment sequence. The number of clusters *K*_*s*_ in each treatment sequence *s* = 1,…,*S* is found by calculating *K*_*s*_ = *Kp*_*s*_ and rounding off. The illustrative example showed how this can be done for a relatively large number of clusters *K* = 33. In cluster randomized stepped wedge designs the number of clusters is often much smaller and often even less than ten. It is then advocated to represent the clusters by fixed effects (i.e. using dummy variables) rather than random effects [21]. As the optimal allocation should be derived using the model that is used to analyze the data, the results in this contribution are not applicable in the case of small number of clusters. For small number of clusters one may wish to derive the optimal number of clusters directly, rather than rounding off cluster sizes as obtained from a continuous optimal design. The design is then a so-called discrete optimal design. A simple approach to finding a discrete optimal design is using brute force: evaluating all possible designs and evaluating which results in lowest $var(\hat{\theta })$. However, the optimization problem becomes more difficult and hence time consuming when the number of sequences and/or the number of clusters increases. Future research may focus on efficient discrete constrained optimization procedures to derive discrete optimal allocations for small number of clusters.

In this paper it was assumed that clusters or (in the case of a cohort design) subjects do not drop out during the course of the study. The stepped wedge design is a longitudinal trials design, and in such designs attrition is the rule rather than the exception. For the individually randomized stepped wedge design [34], it has already been shown that attrition may have a large effect on the optimal allocation and also precision of the treatment effect estimator. For large values of the individual autocorrelation coefficient the design is uniform in the case attrition is absent. When attrition is present, treatment sequences that have their switch early in time get assigned a larger number of individuals than those that have their switch later in time. Future research should study if such a finding also holds for the cluster randomized stepped wedge design. In addition to that, it may be studied if attrition of clusters has a larger or smaller effect than attrition of individuals. Attrition of subjects in a cohort study results in unequal cluster-period sizes over time. Unequal cluster-period sizes may also occur in cross-sectional studies, for instance when clusters are not able or willing to recruit the same number of subjects within each cluster-period. For instance, clusters may be very eager to participate at the beginning of the trial and be able to recruit the requested amount of subjects in the earlier time periods, but may somewhat lose their interest in the trial later in time and then spend less effort to recruit subjects. It will have to be studied in future research to what extent the optimal allocations in this paper are affected when the trial includes cluster-periods of unequal sizes. Related to this, it will have to be studied to what how the precision is affected when the number of clusters and/or subjects deviates from the plan.

Another direction of future research is the derivation of optimal allocations under treatment effect heterogeneity [35, 36]. In such designs the effect of treatment varies across the clusters and an extra random effect is needed in the multilevel model to allow for treatment effect heterogeneity. Furthermore, I would expect optimal allocations to become different from those presented here when the treatment effect is non-sustainable, meaning it diminishes over time after the treatment switch. It would also be of interest to verify if the optimal allocations still hold if time is not represented by dummies, but by some trend, such as a linear or quadratic growth model.

To my knowledge this was the first study on optimal allocation of clusters to sequences in stepped wedge designs for the recently introduced exponential decay correlation structure. I hope the results in this paper, along with the R script on my GitHub page, enables to design their trial in an efficient way, and to convince them not always to use the uniform allocation.

## Supporting information

S1 Appendix Extensive results for the cross-sectional design. (PDF)

S2 Appendix Extensive results for the cohort design. (PDF)

S3 Appendix Power book 2016 table 11.1 for PLOS ONE. (PDF)
