# Supplementary material for: Optimal allocation of clusters in stepped wedge designs with a decaying correlation structure
Source: PLoS One. 2023 Aug 16;18(8):e0289275. doi: 10.1371/journal.pone.0289275 (PMC10431648; doi:10.1371/journal.pone.0289275)

Number of sequences $S = 3$

Intraclass correlation $\rho = 0.0125$

Number of subjects per cluster-period $m = 5$

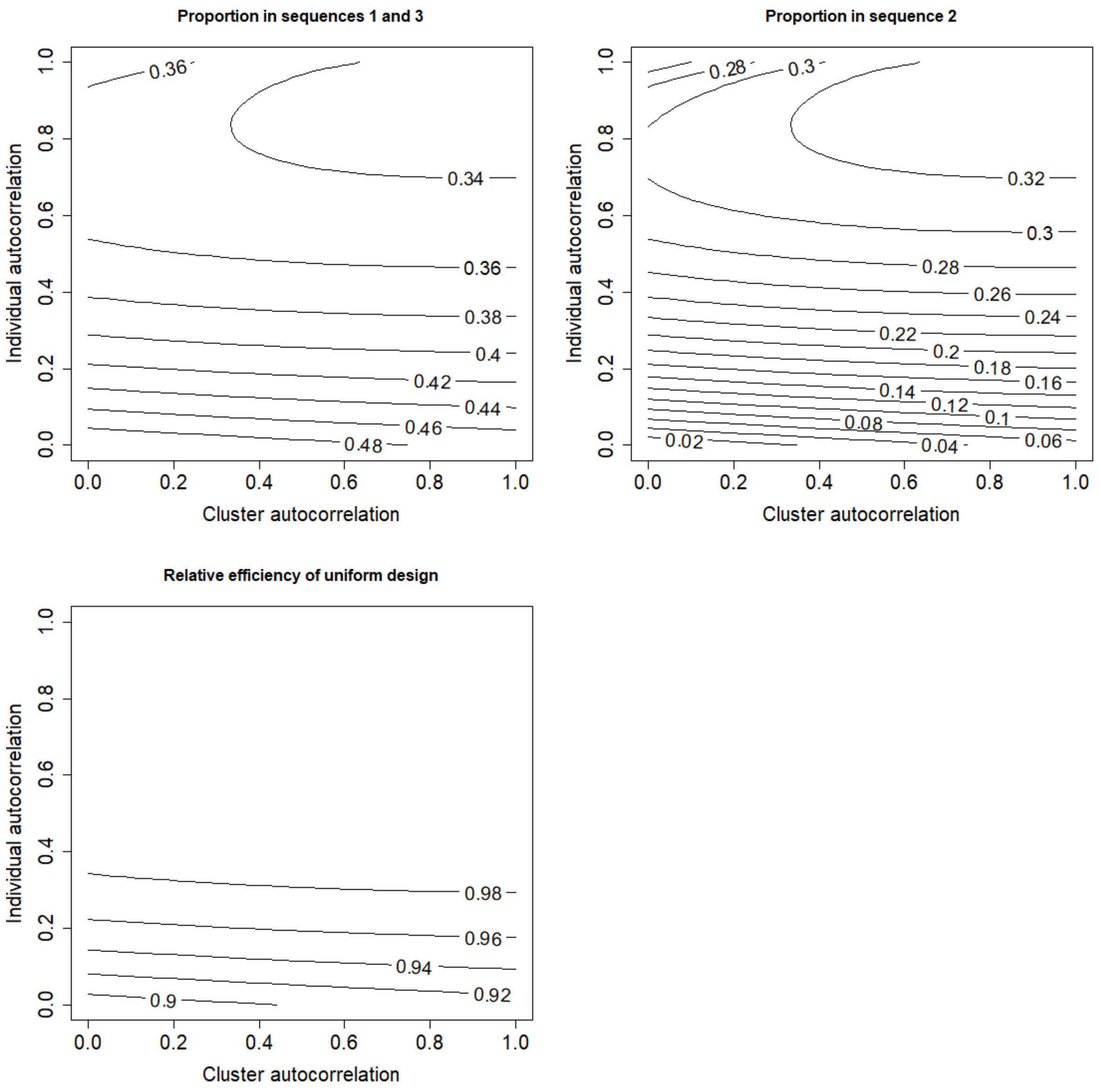

Number of sequences $S = 3$

Intraclass correlation $\rho = 0.025$

Number of subjects per cluster-period $m = 5$

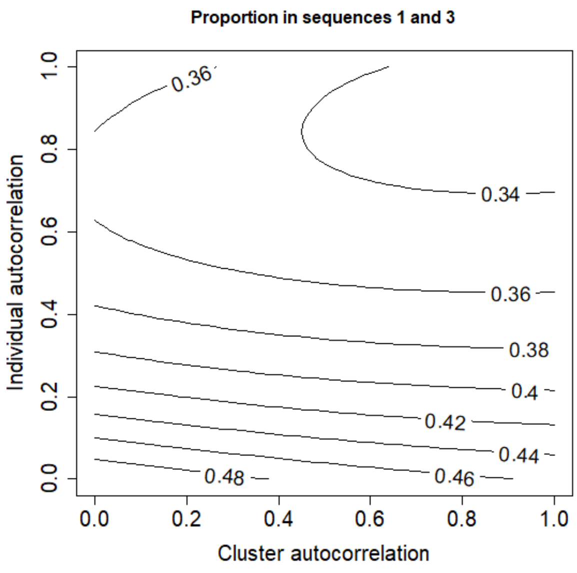

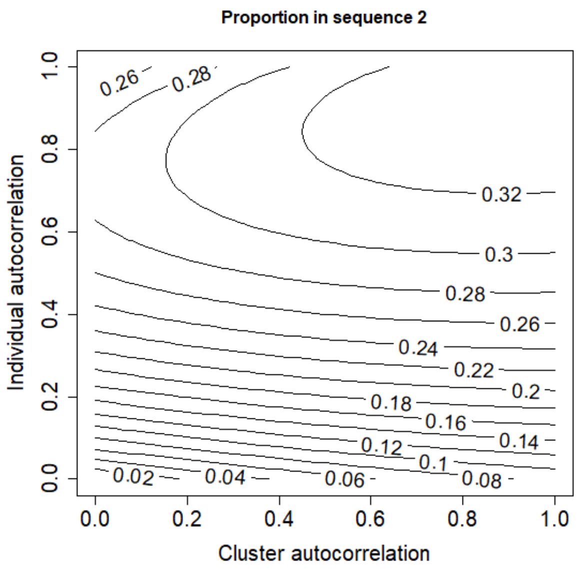

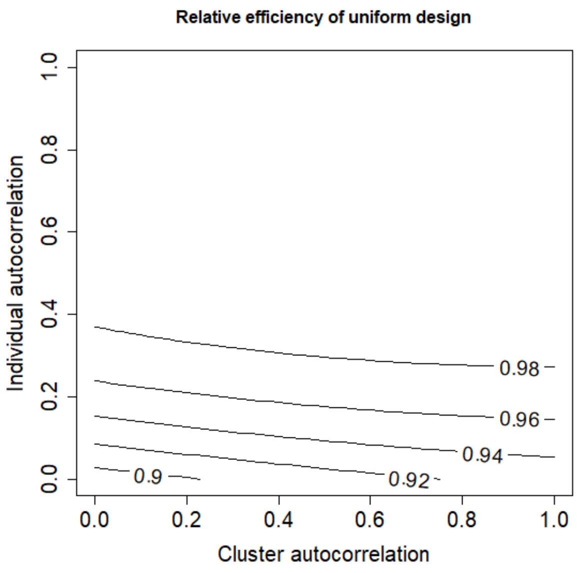

Number of sequences $S = 3$

Intraclass correlation $\rho = 0.05$

Number of subjects per cluster-period $m = 5$

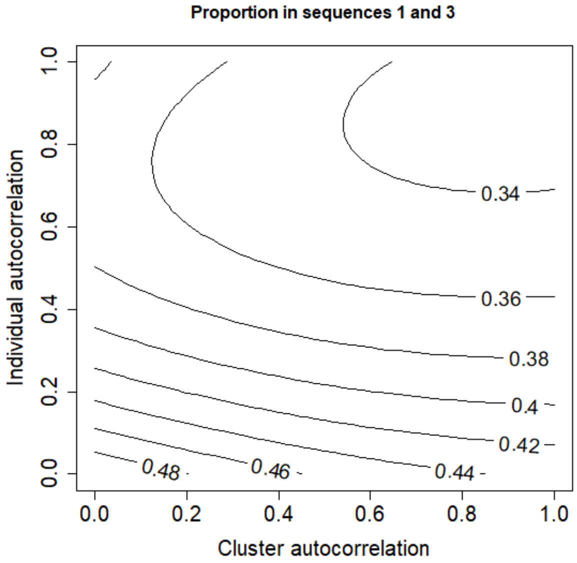

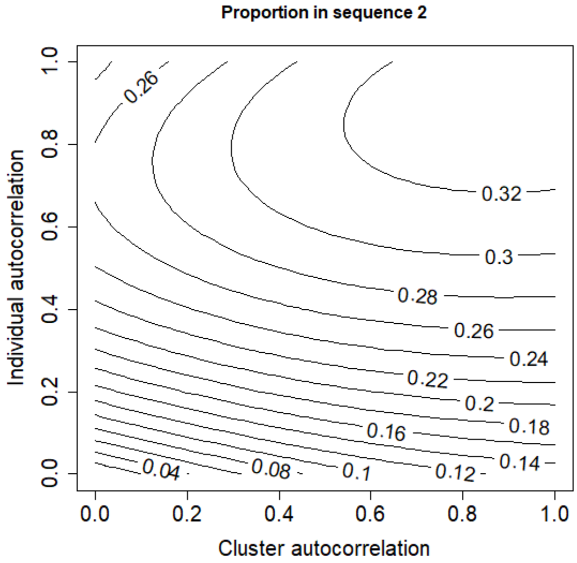

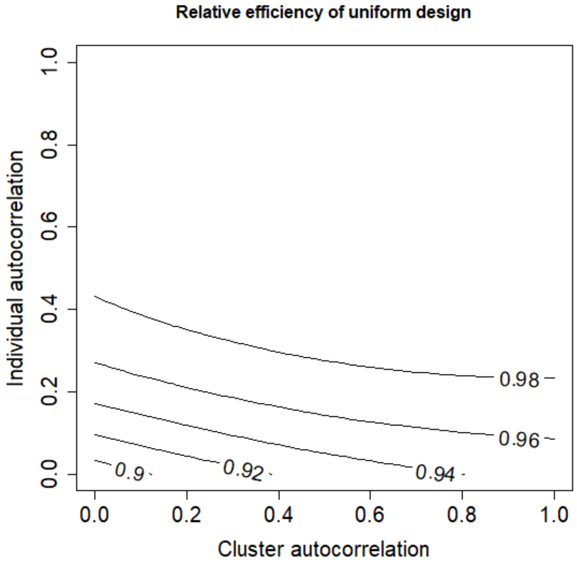

Number of sequences $S = 3$

Intraclass correlation $\rho = 0.0125$

Number of subjects per cluster-period $m = 25$

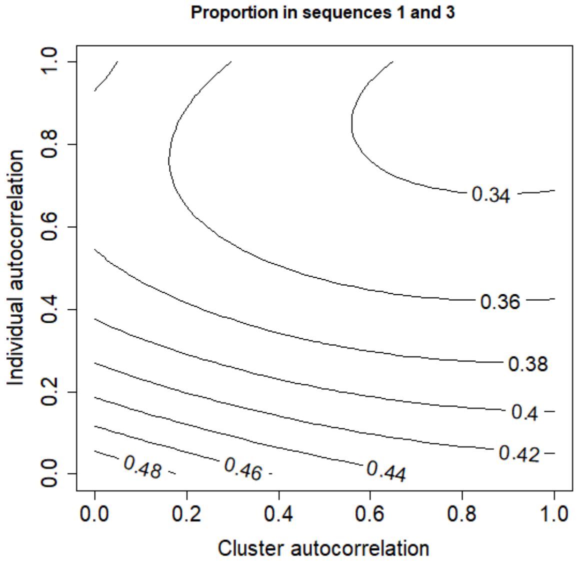

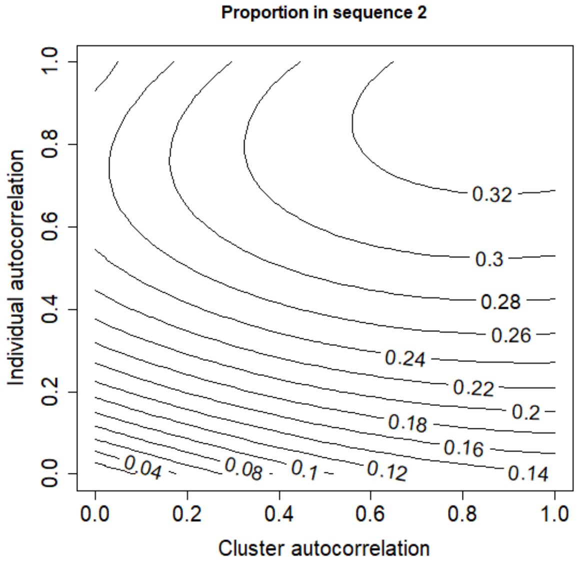

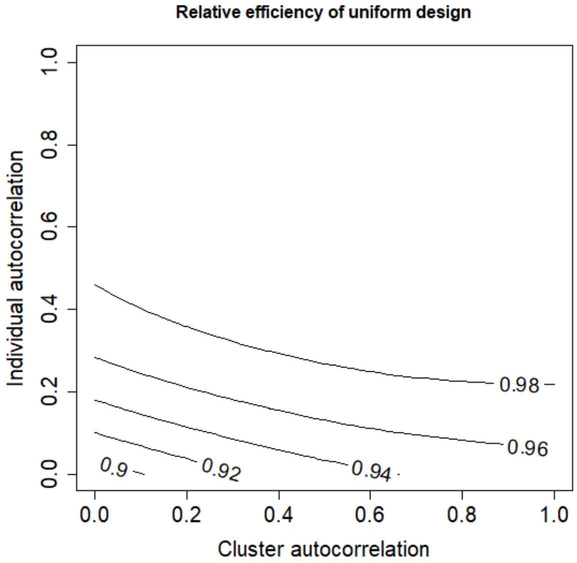

Number of sequences $S = 3$

Intraclass correlation $\rho = 0.025$

Number of subjects per cluster-period $m = 25$

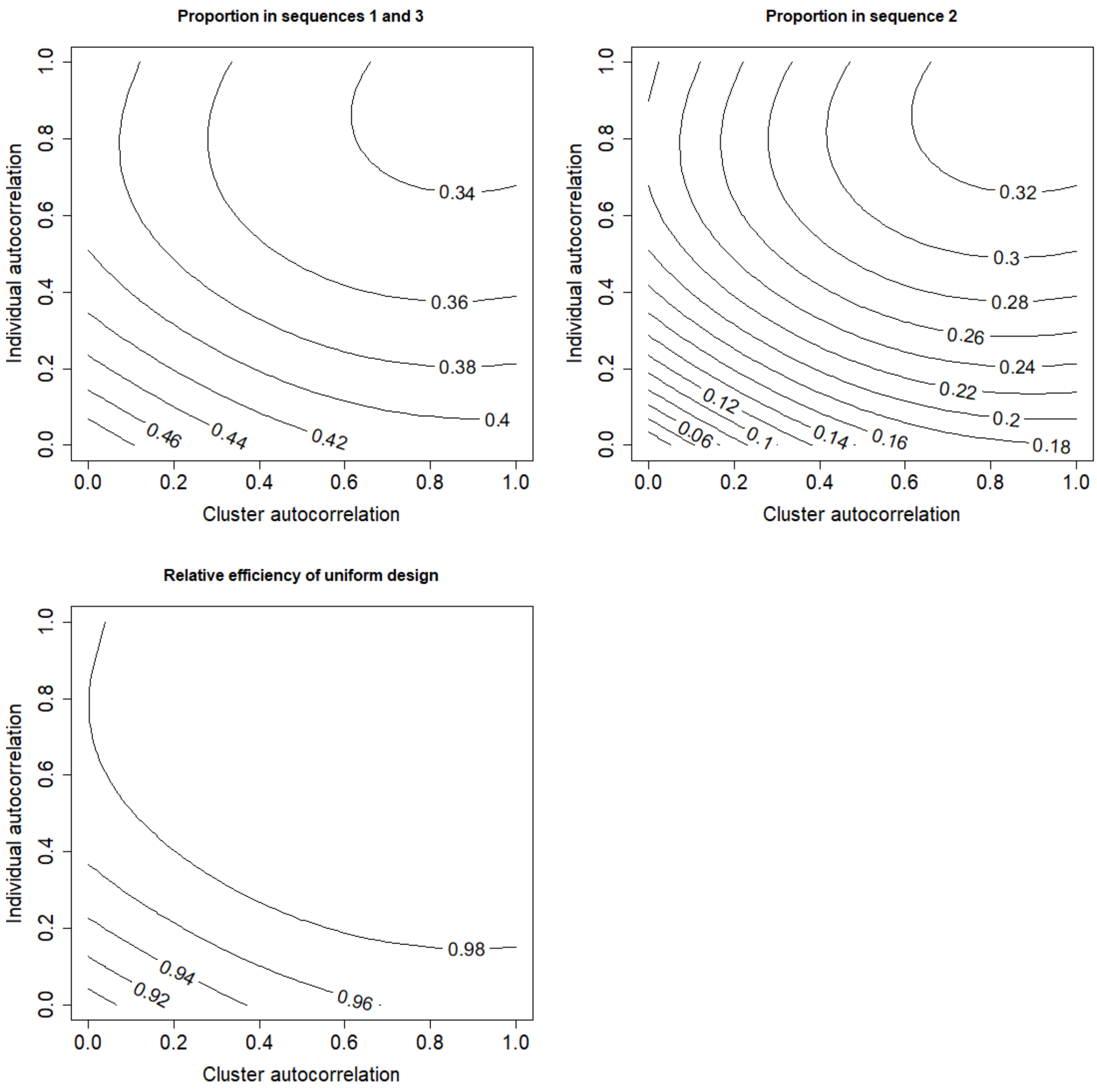

Number of sequences $S = 3$

Intraclass correlation $\rho = 0.05$

Number of subjects per cluster-period $m = 25$

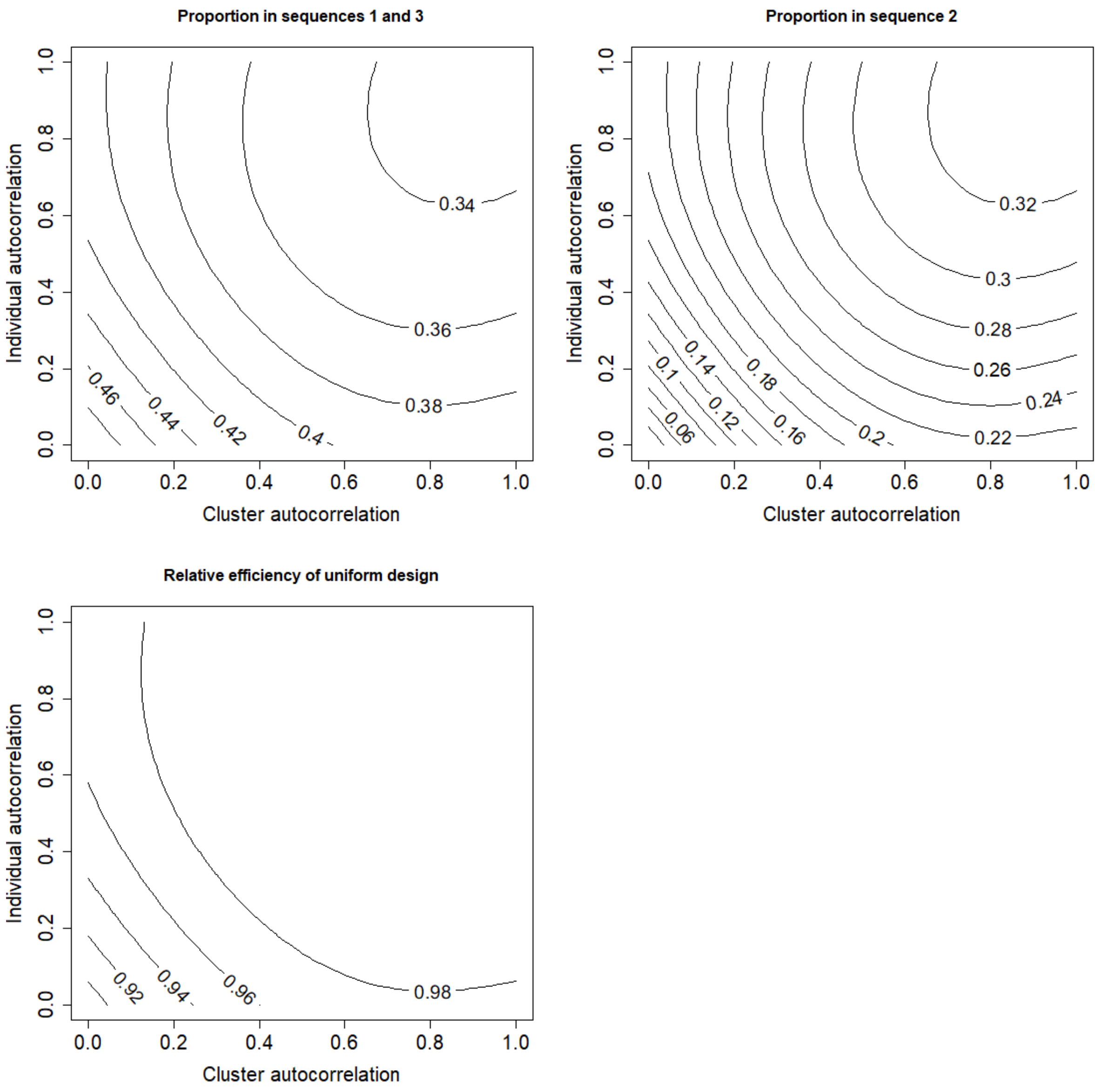

Number of sequences $S = 3$

Intraclass correlation $\rho = 0.0125$

Number of subjects per cluster-period $m = 50$

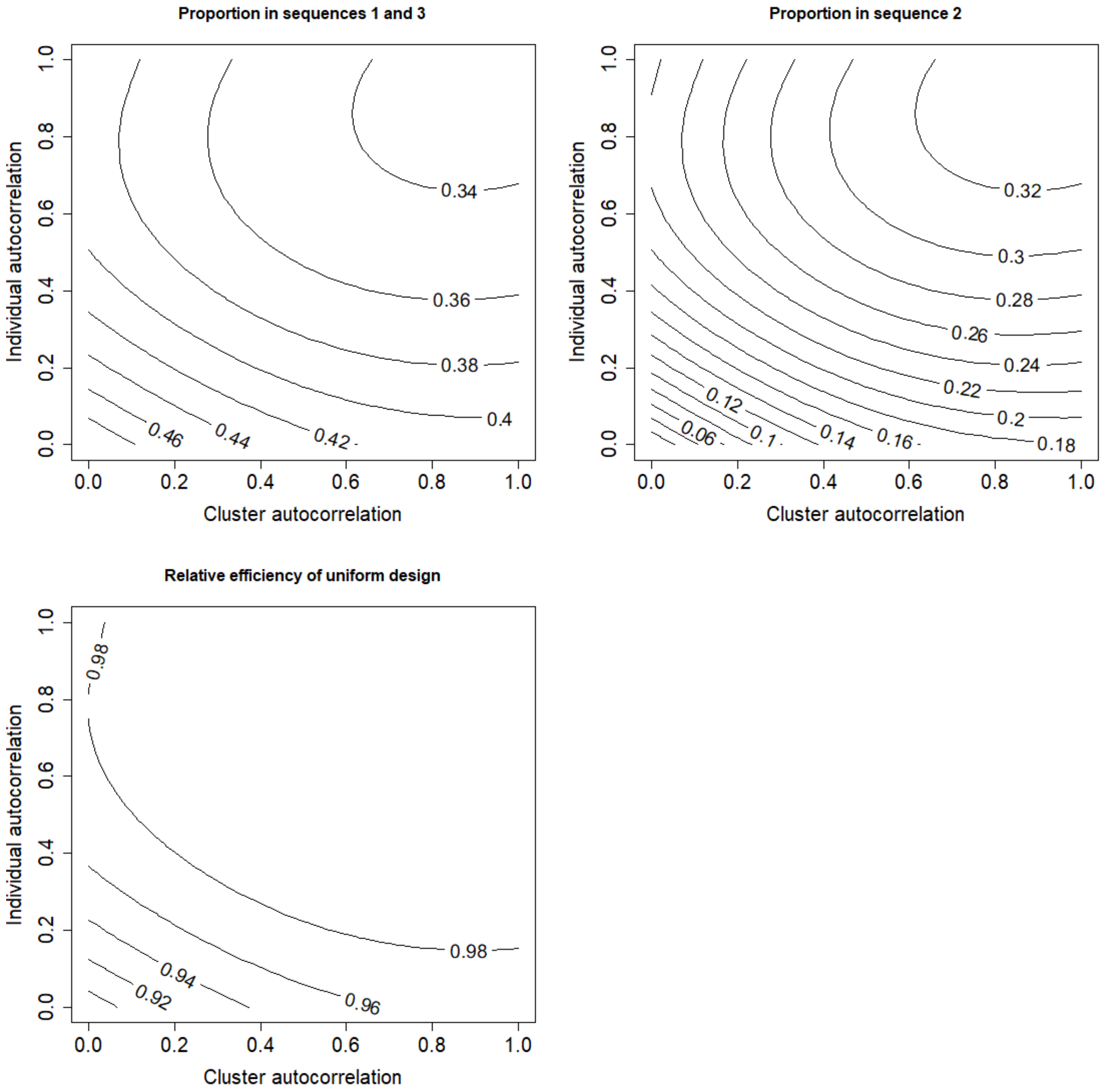

Number of sequences $S = 3$

Intraclass correlation $\rho = 0.025$

Number of subjects per cluster-period $m = 50$

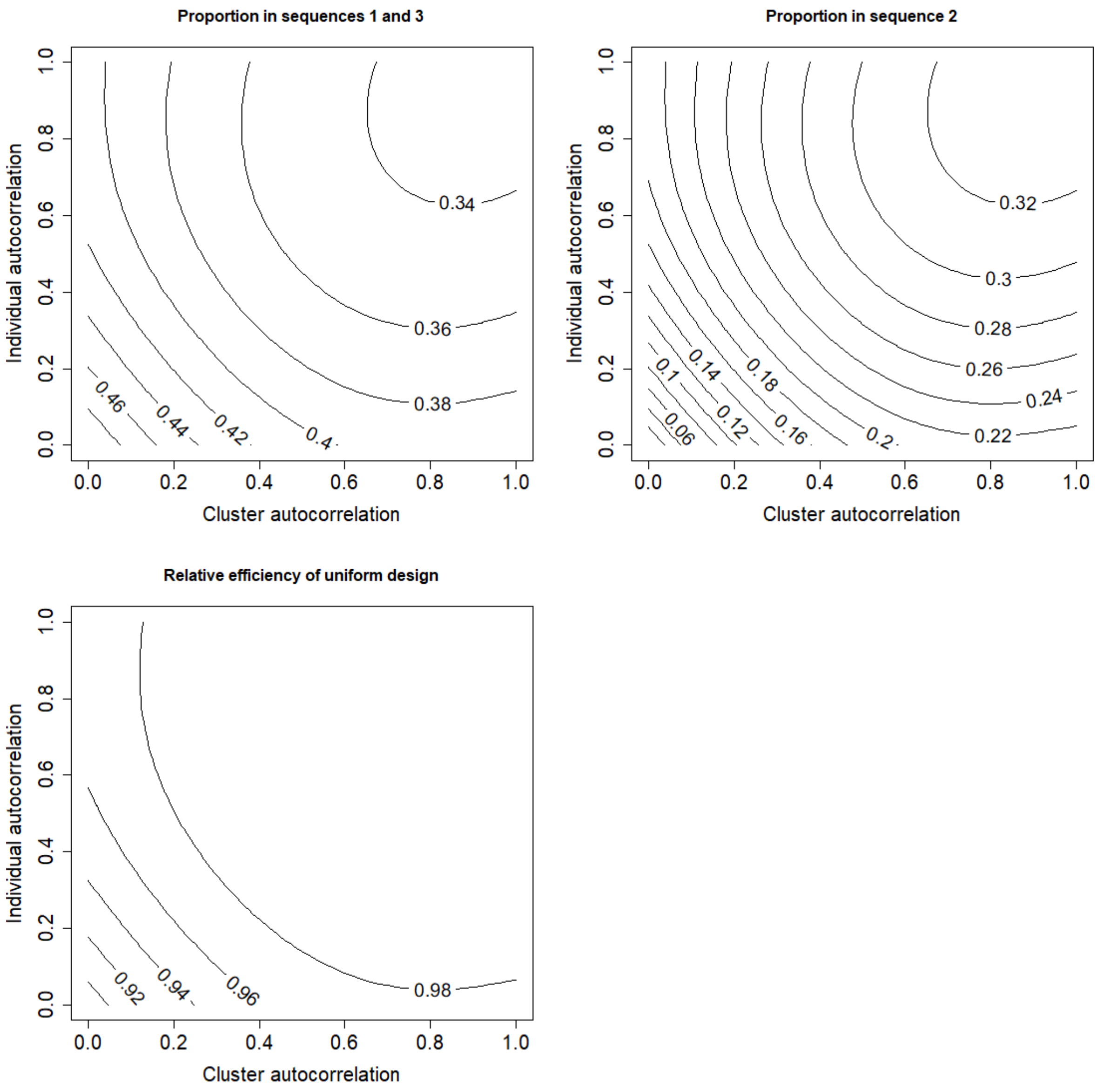

Number of sequences $S = 3$

Intraclass correlation $\rho = 0.05$

Number of subjects per cluster-period $m = 50$

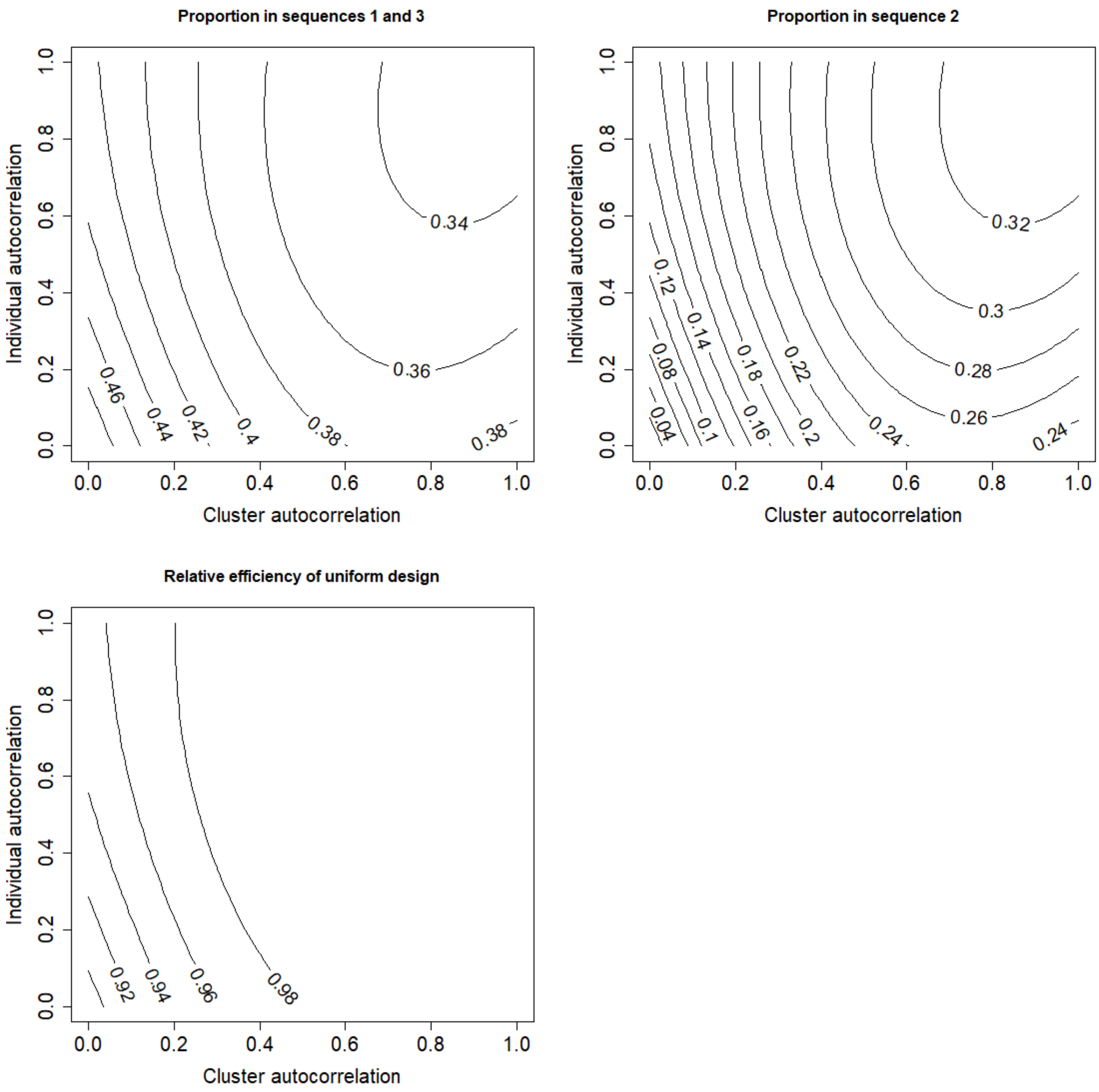

Number of sequences $S = 4$

Intraclass correlation $\rho = 0.0125$

Number of subjects per cluster-period $m = 5$

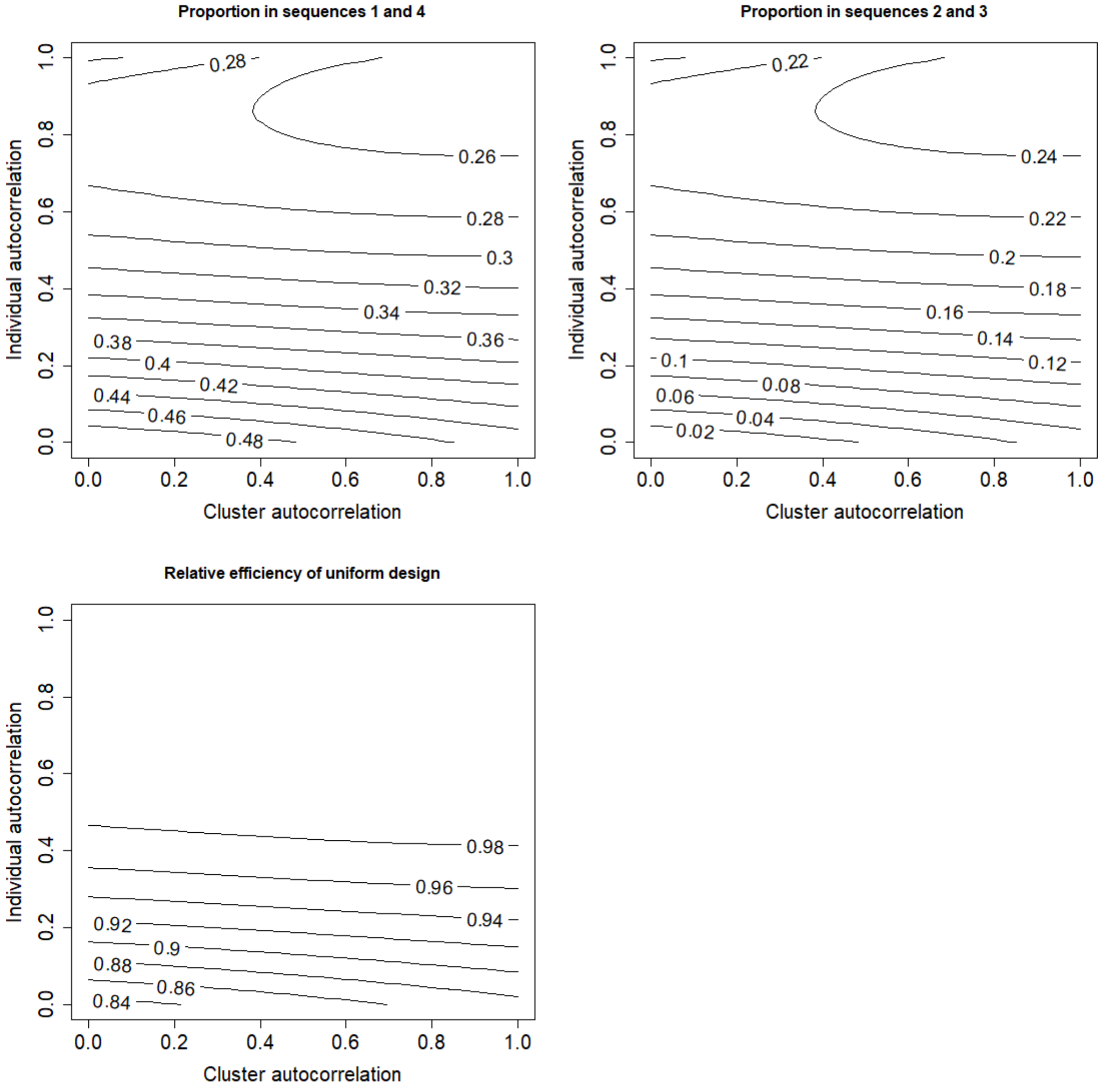

Number of sequences $S = 4$

Intraclass correlation $\rho = 0.025$

Number of subjects per cluster-period $m = 5$

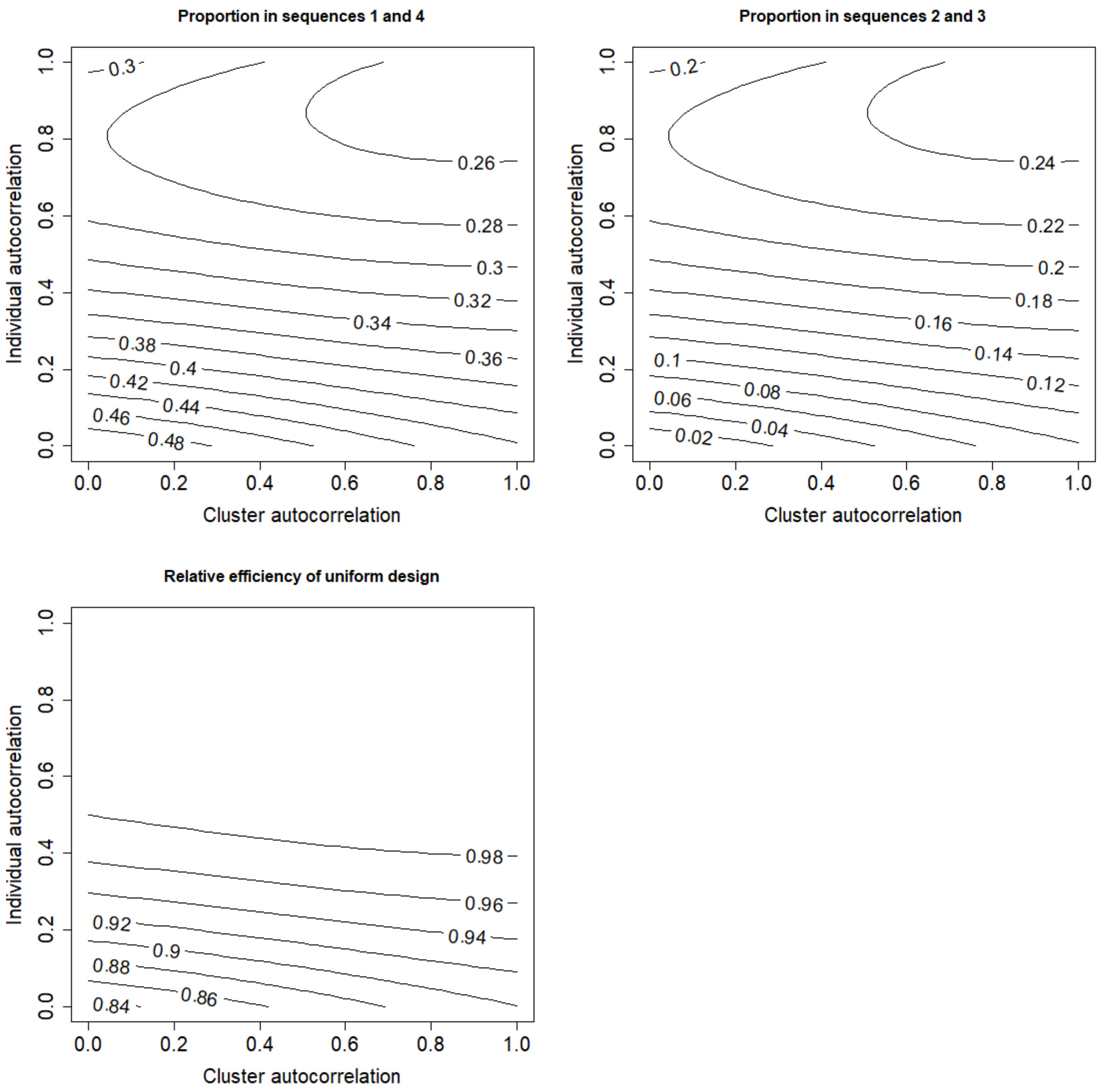

Number of sequences	$S = 4$
Intraclass correlation	$\rho = 0.05$
Number of subjects per cluster-period	$m = 5$

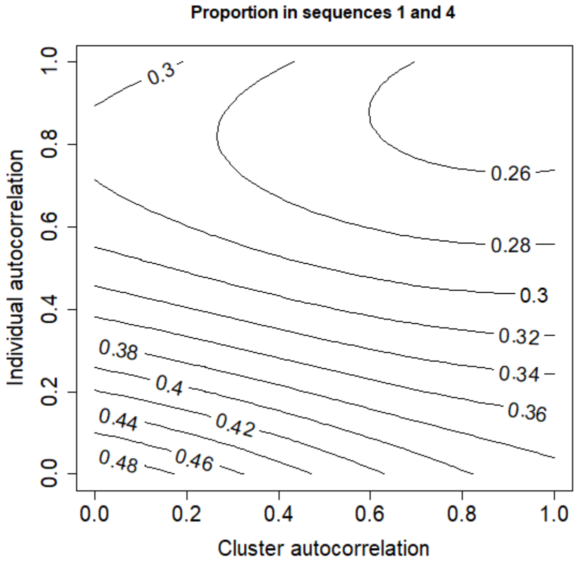

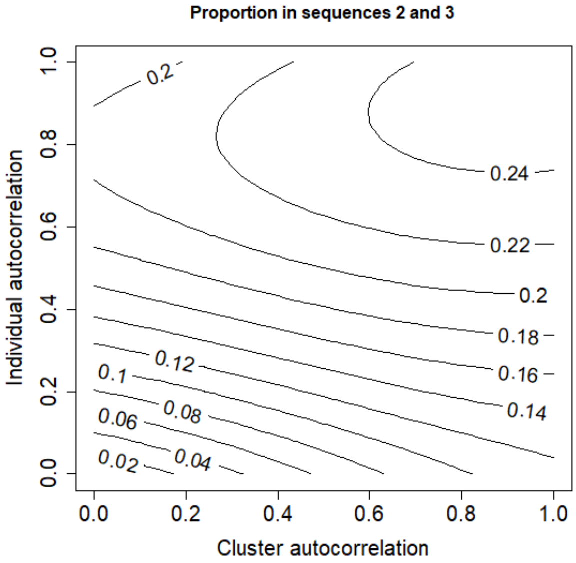

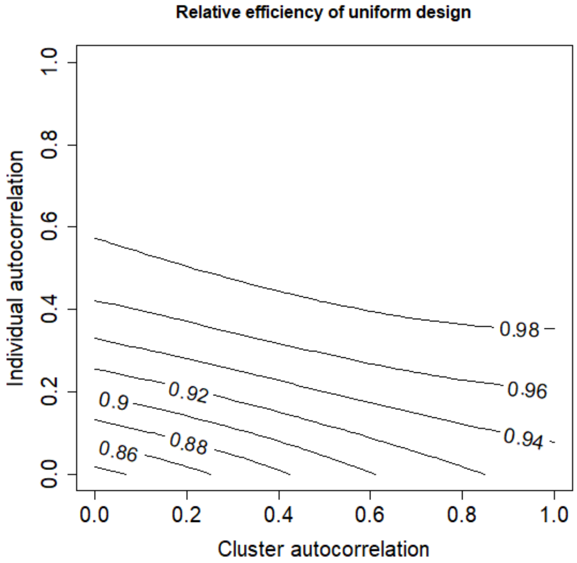

Number of sequences $S = 4$

Intraclass correlation $\rho = 0.0125$

Number of subjects per cluster-period $m = 25$

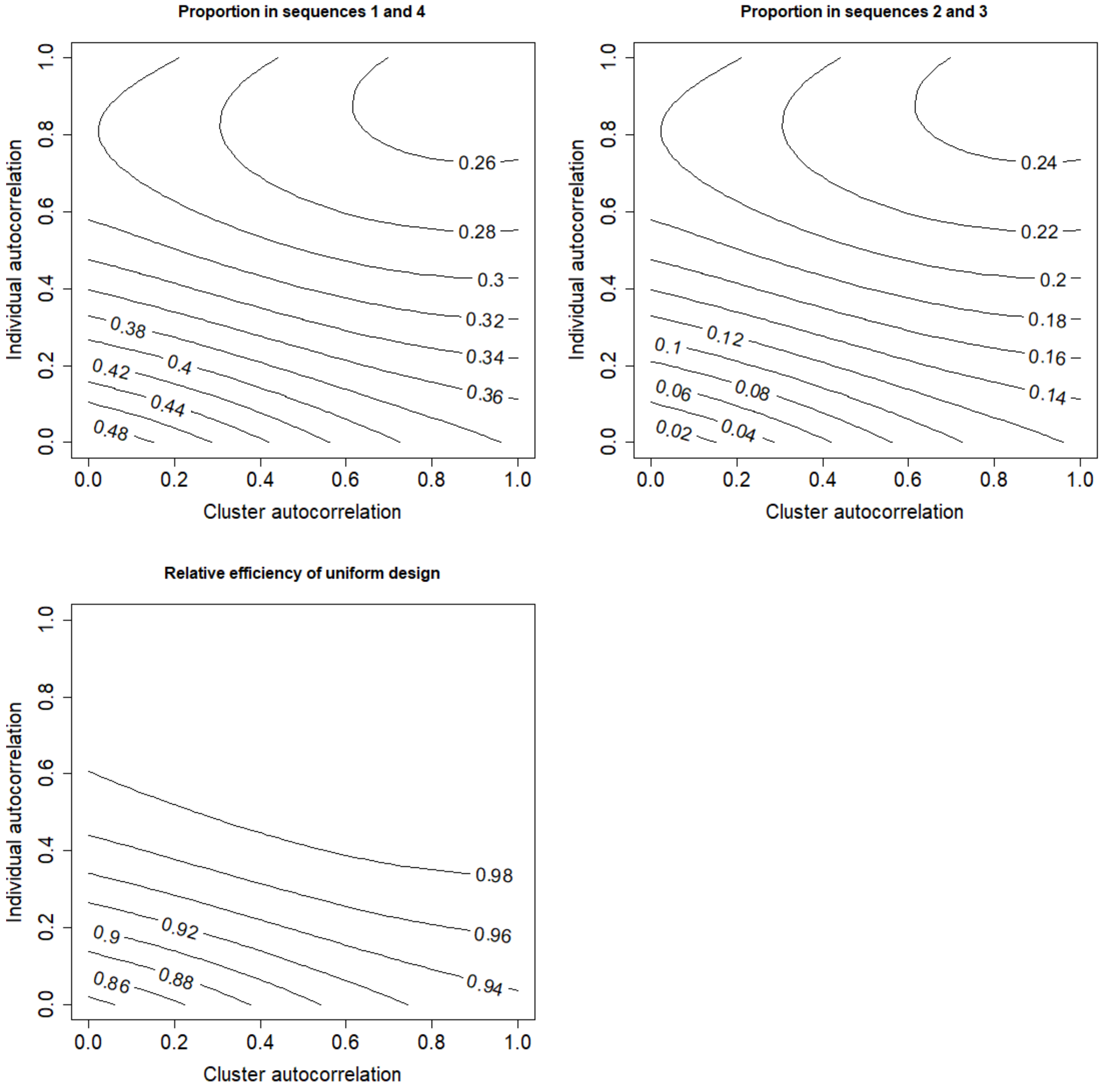

Number of sequences $S = 4$

Intraclass correlation $\rho = 0.025$

Number of subjects per cluster-period $m = 25$

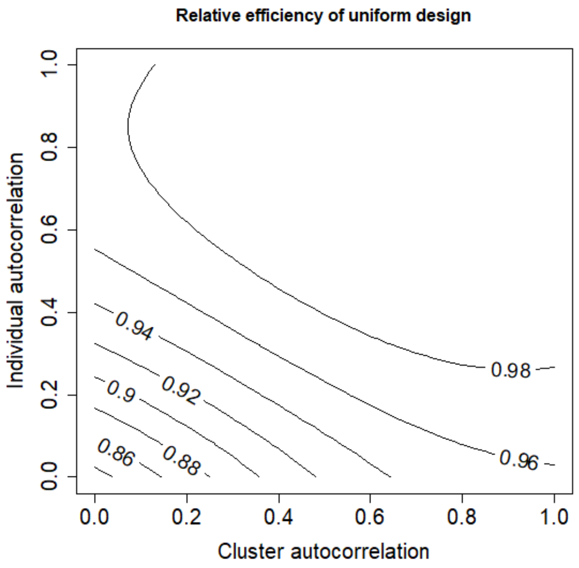

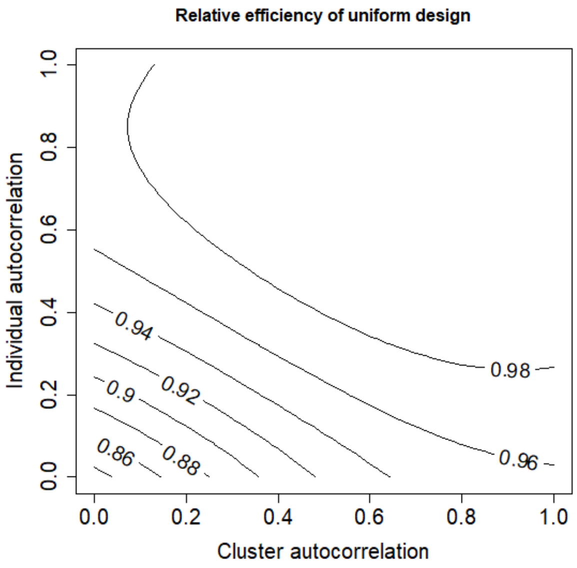

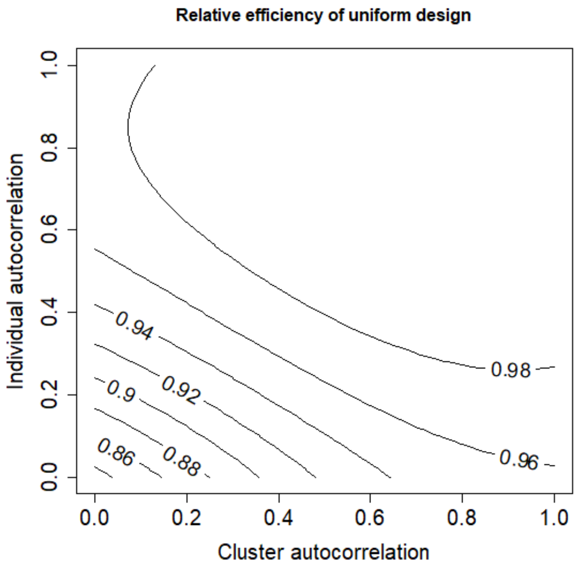

Number of sequences $S = 4$

Intraclass correlation $\rho = 0.05$

Number of subjects per cluster-period $m = 25$

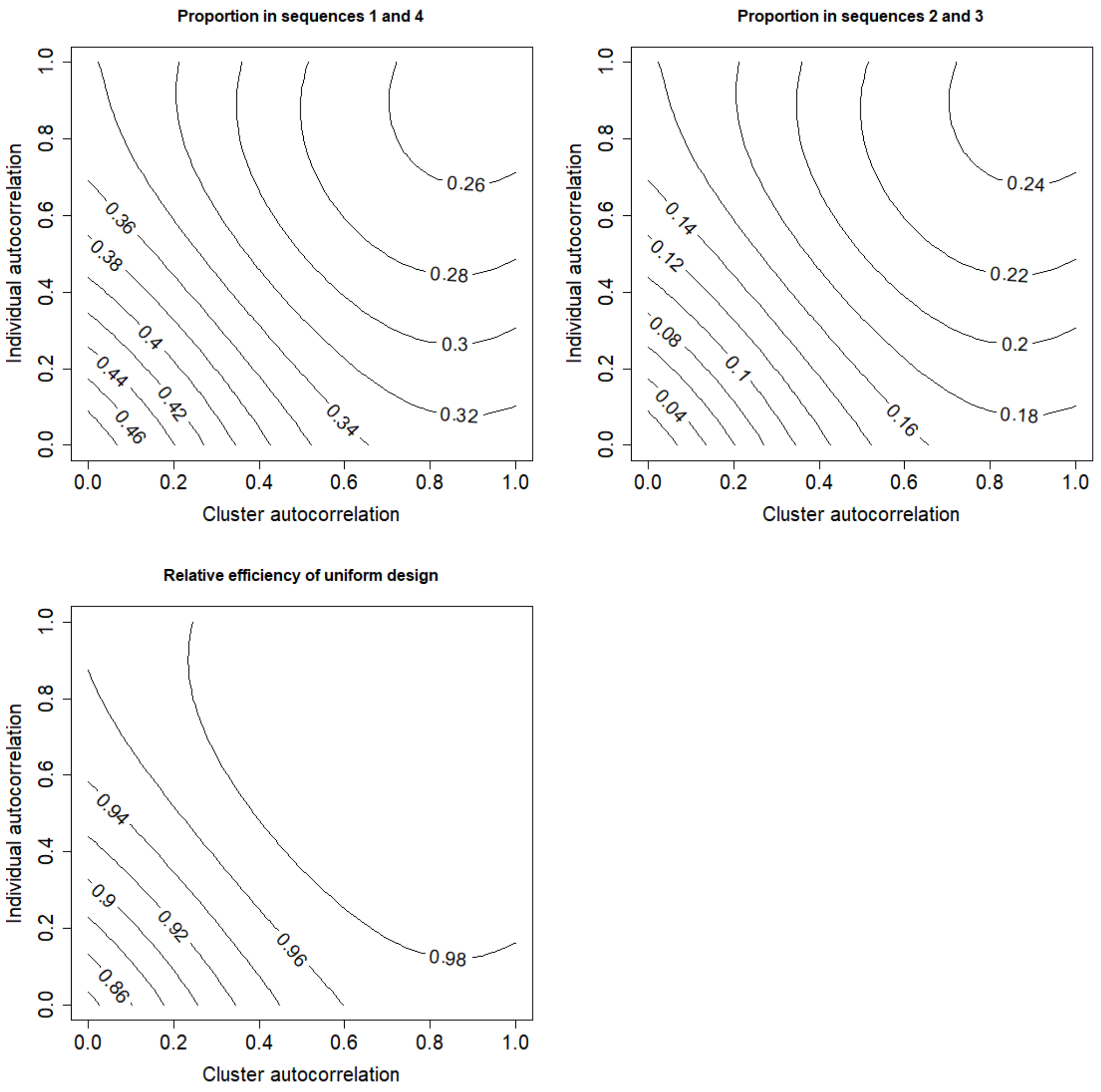

Number of sequences $S = 4$

Intraclass correlation $\rho = 0.0125$

Number of subjects per cluster-period $m = 50$

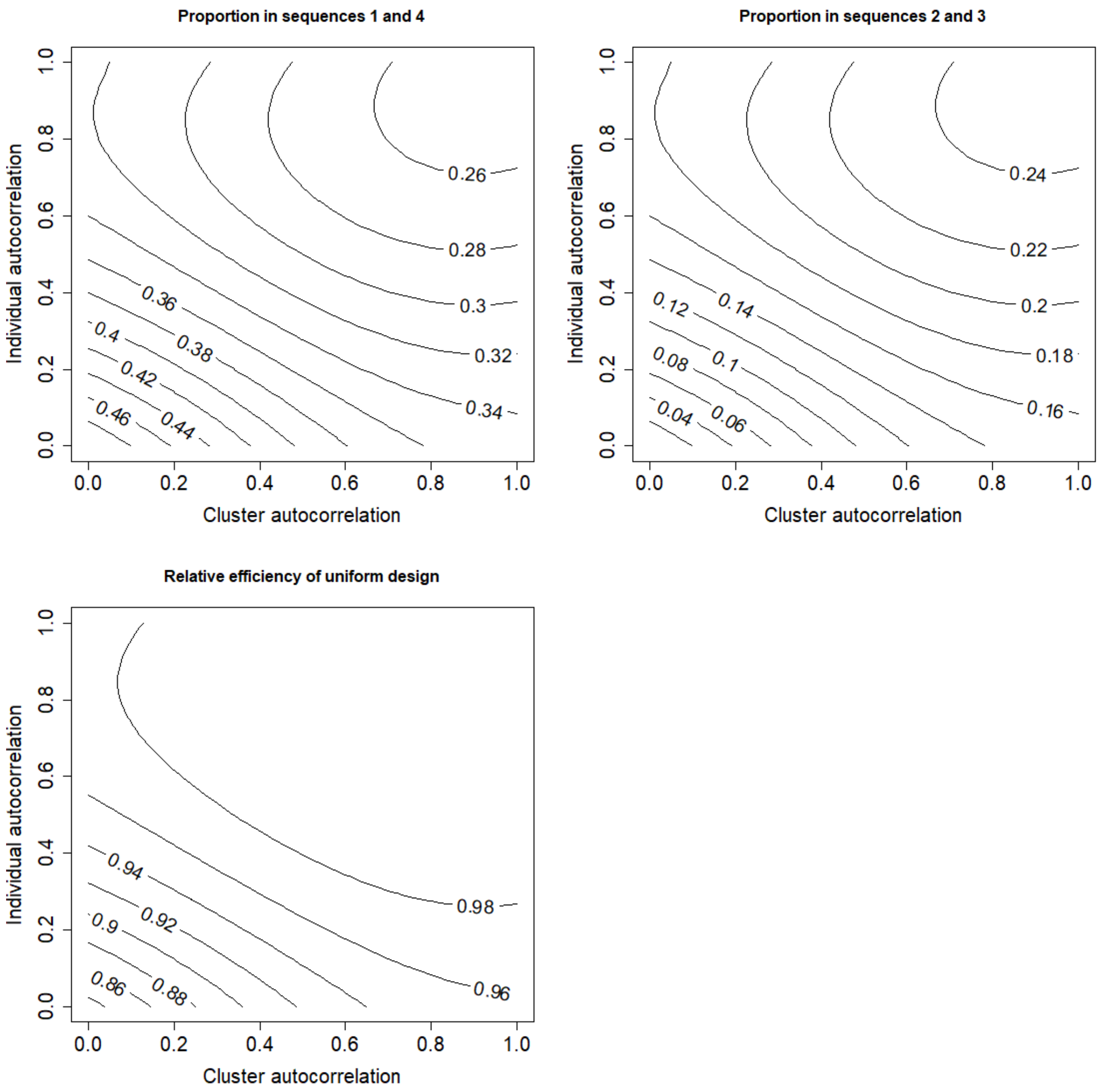

Number of sequences $S = 4$

Intraclass correlation $\rho = 0.025$

Number of subjects per cluster-period $m = 50$

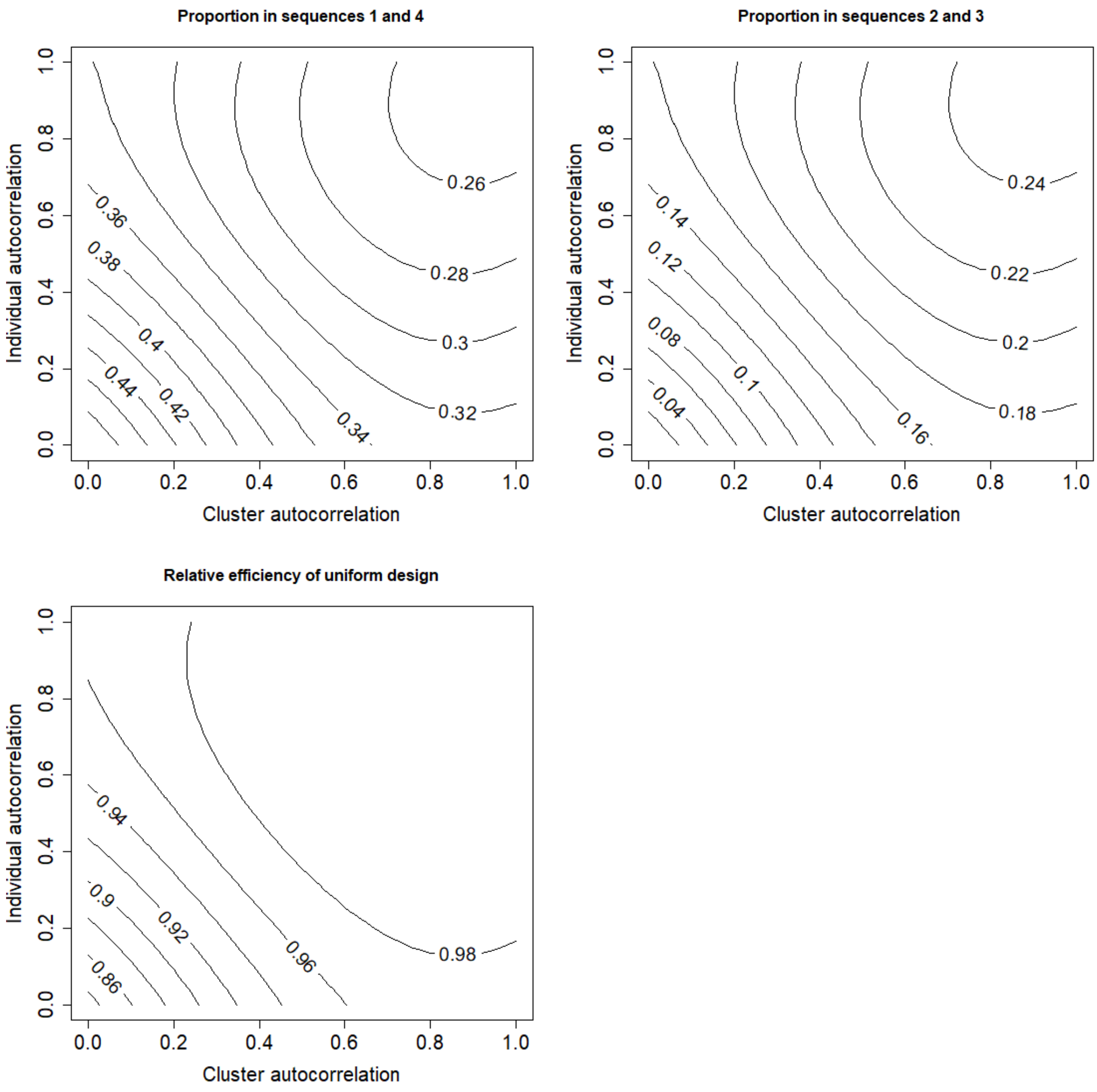

Number of sequences $S = 4$

Intraclass correlation $\rho = 0.05$

Number of subjects per cluster-period $m = 50$

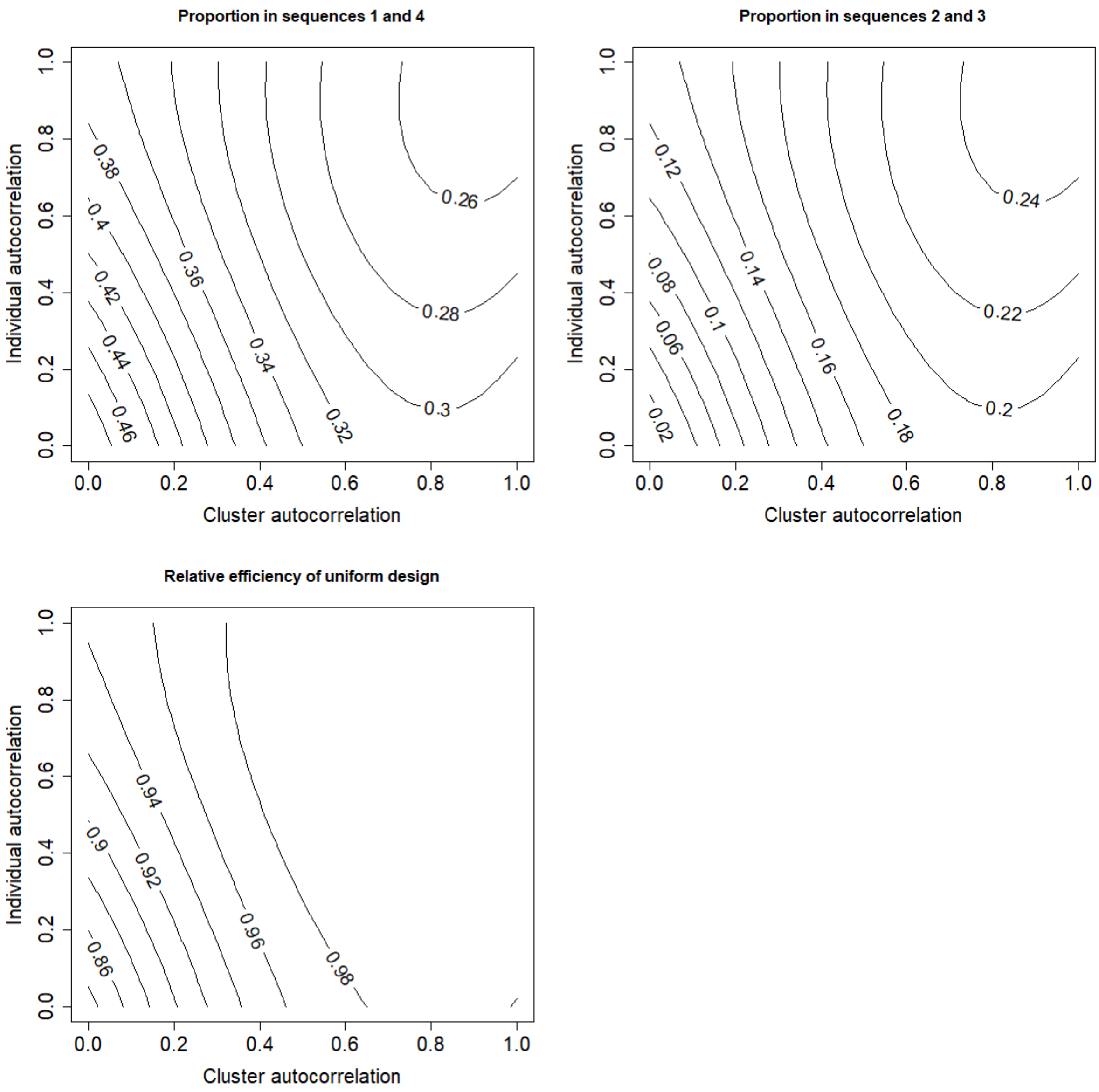

Number of sequences $S = 5$

Intraclass correlation $\rho = 0.0125$

Number of subjects per cluster-period $m = 5$

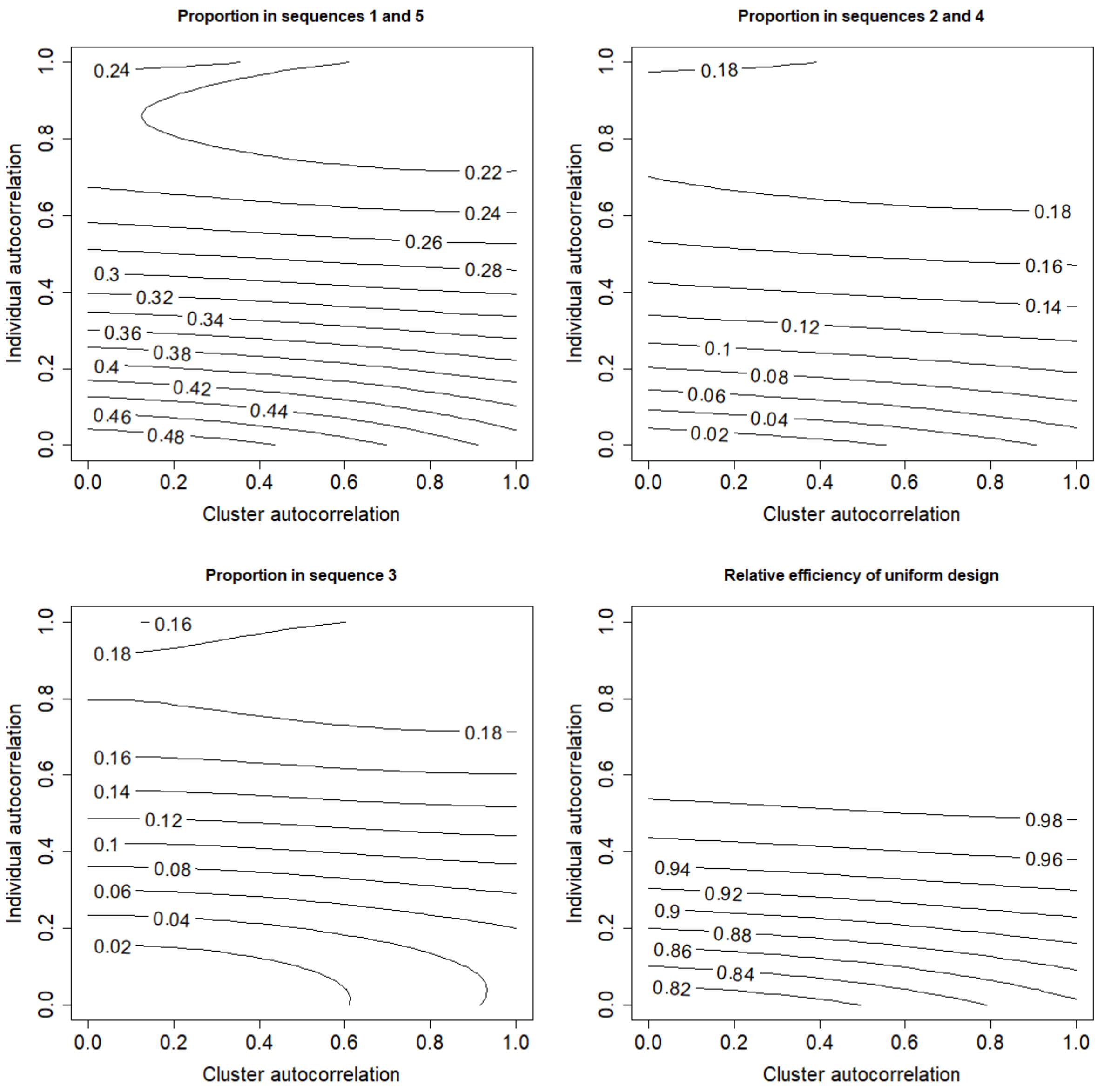

Number of sequences $S = 5$

Intraclass correlation $\rho = 0.025$

Number of subjects per cluster-period $m = 5$

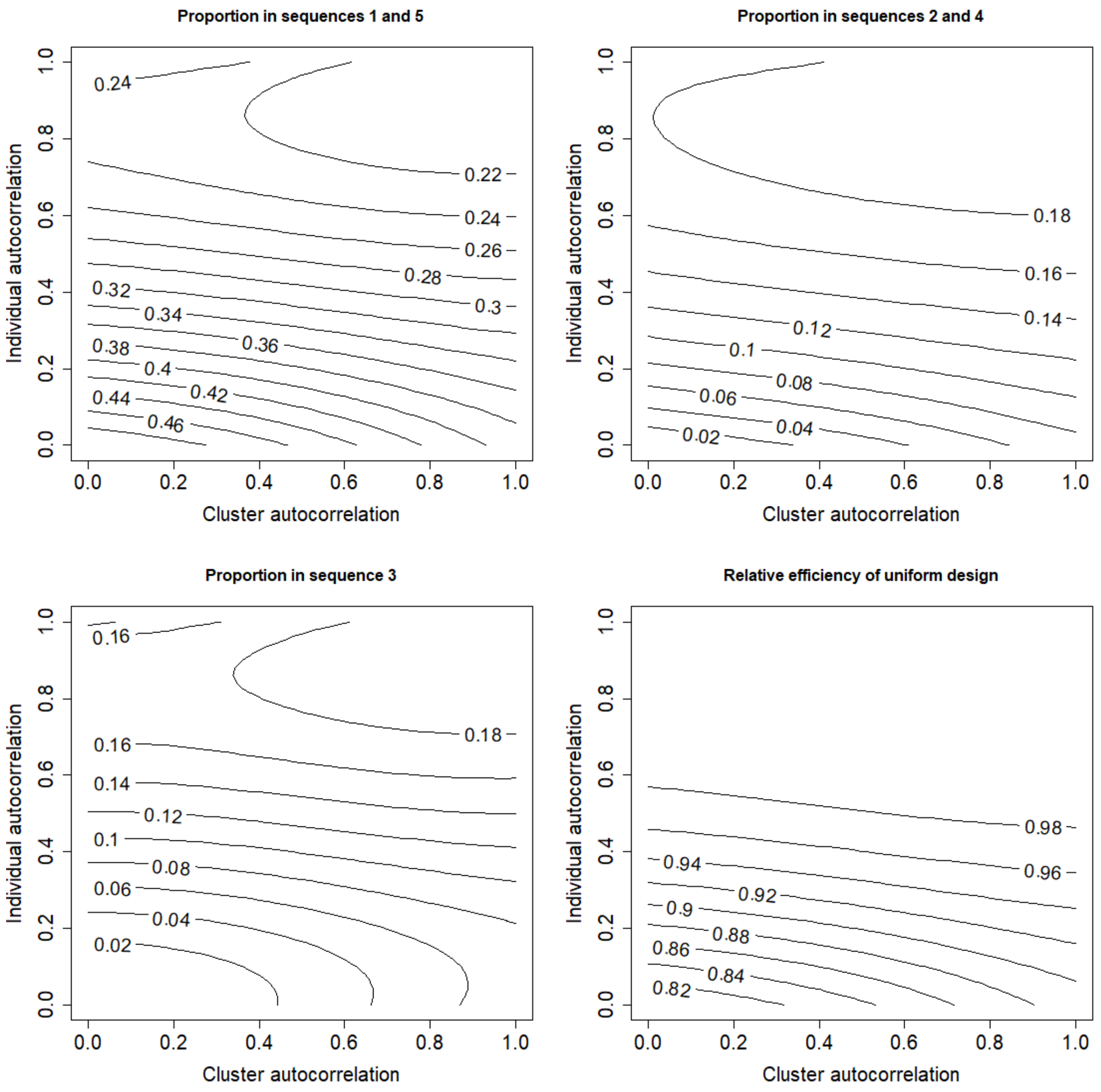

Number of sequences $S = 5$

Intraclass correlation $\rho = 0.05$

Number of subjects per cluster-period $m = 5$

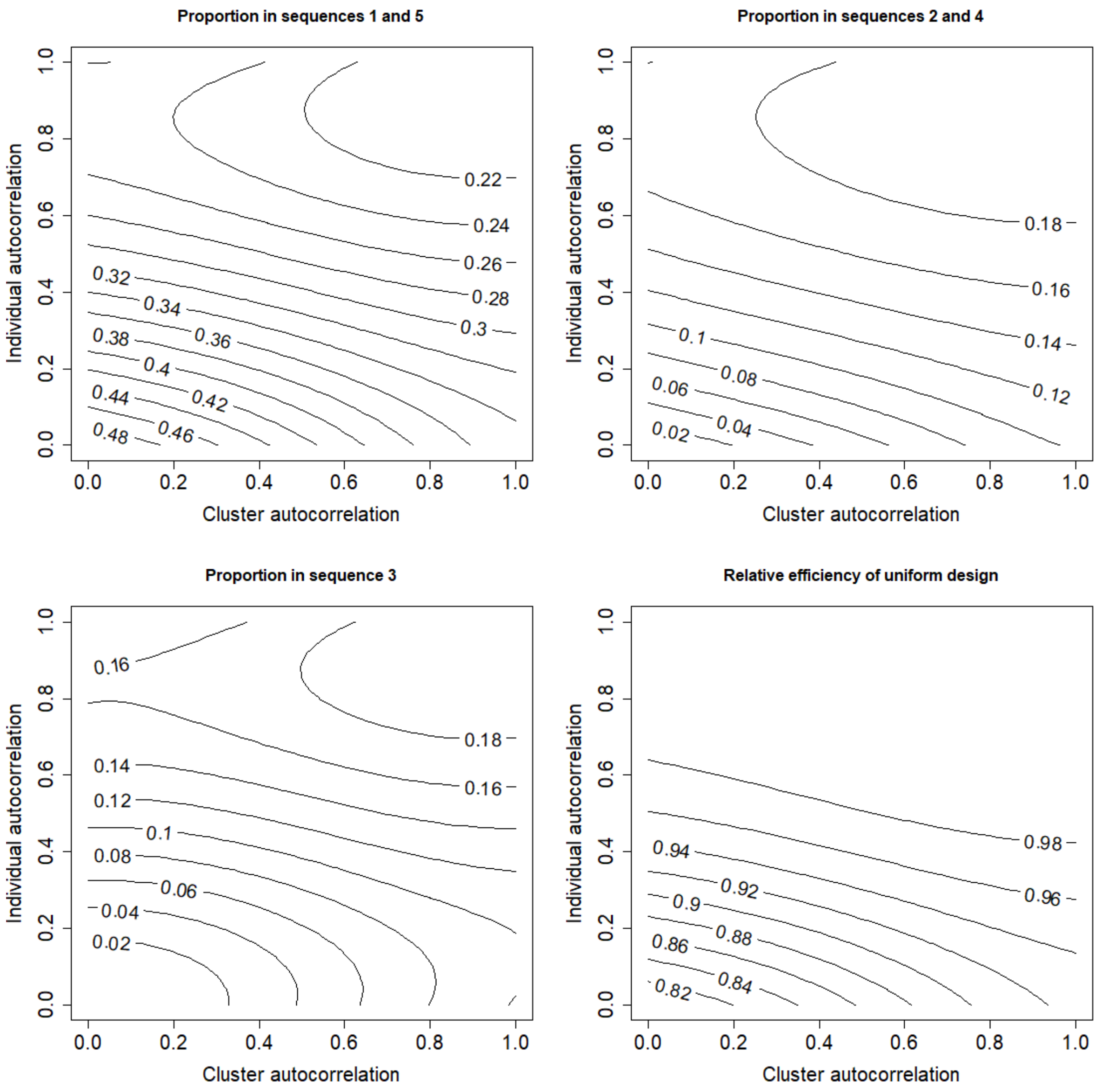

Number of sequences	$S = 5$
Intraclass correlation	$\rho = 0.0125$
Number of subjects per cluster-period	$m = 25$

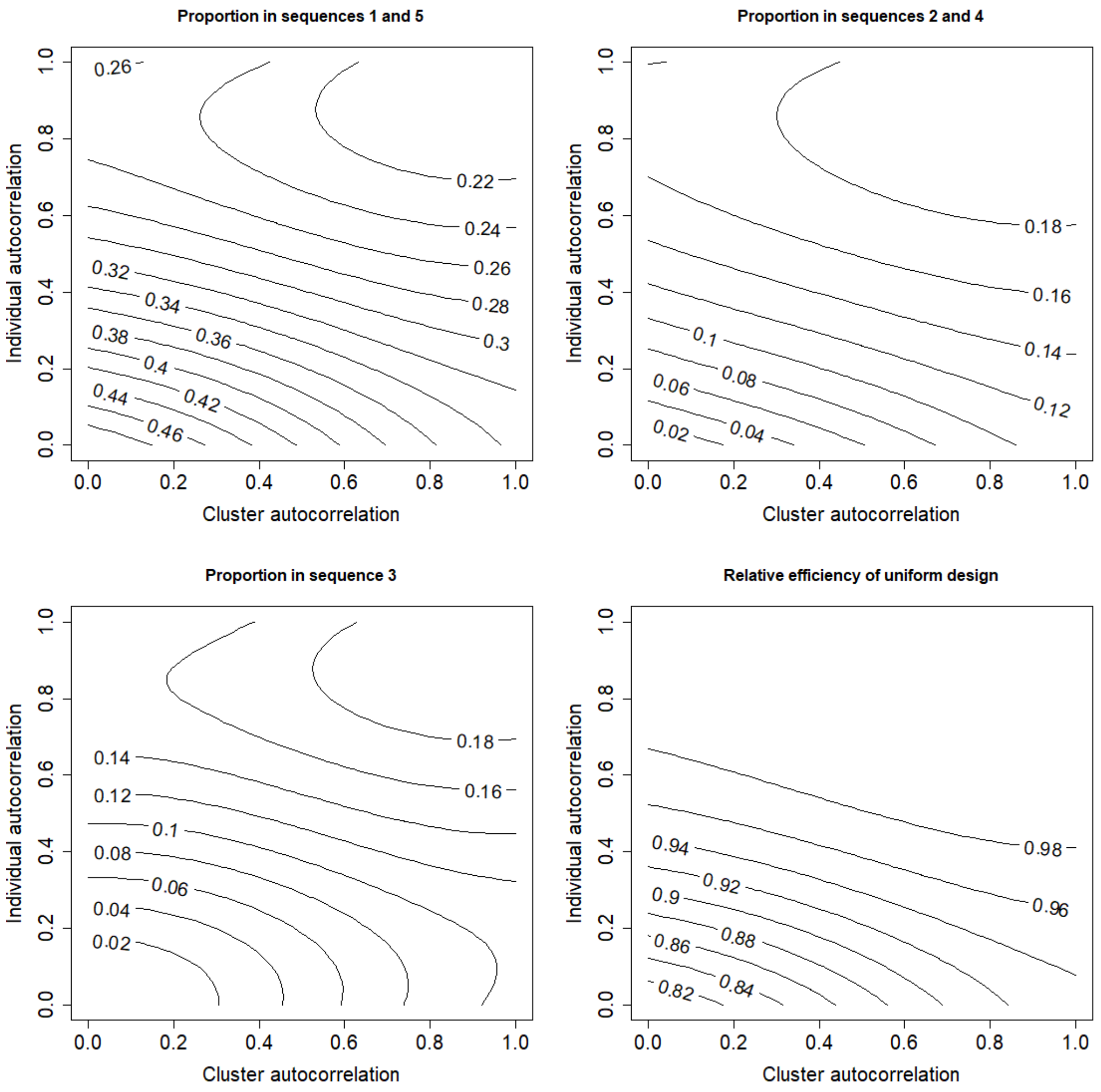

Number of sequences $S = 5$

Intraclass correlation $\rho = 0.025$

Number of subjects per cluster-period $m = 25$

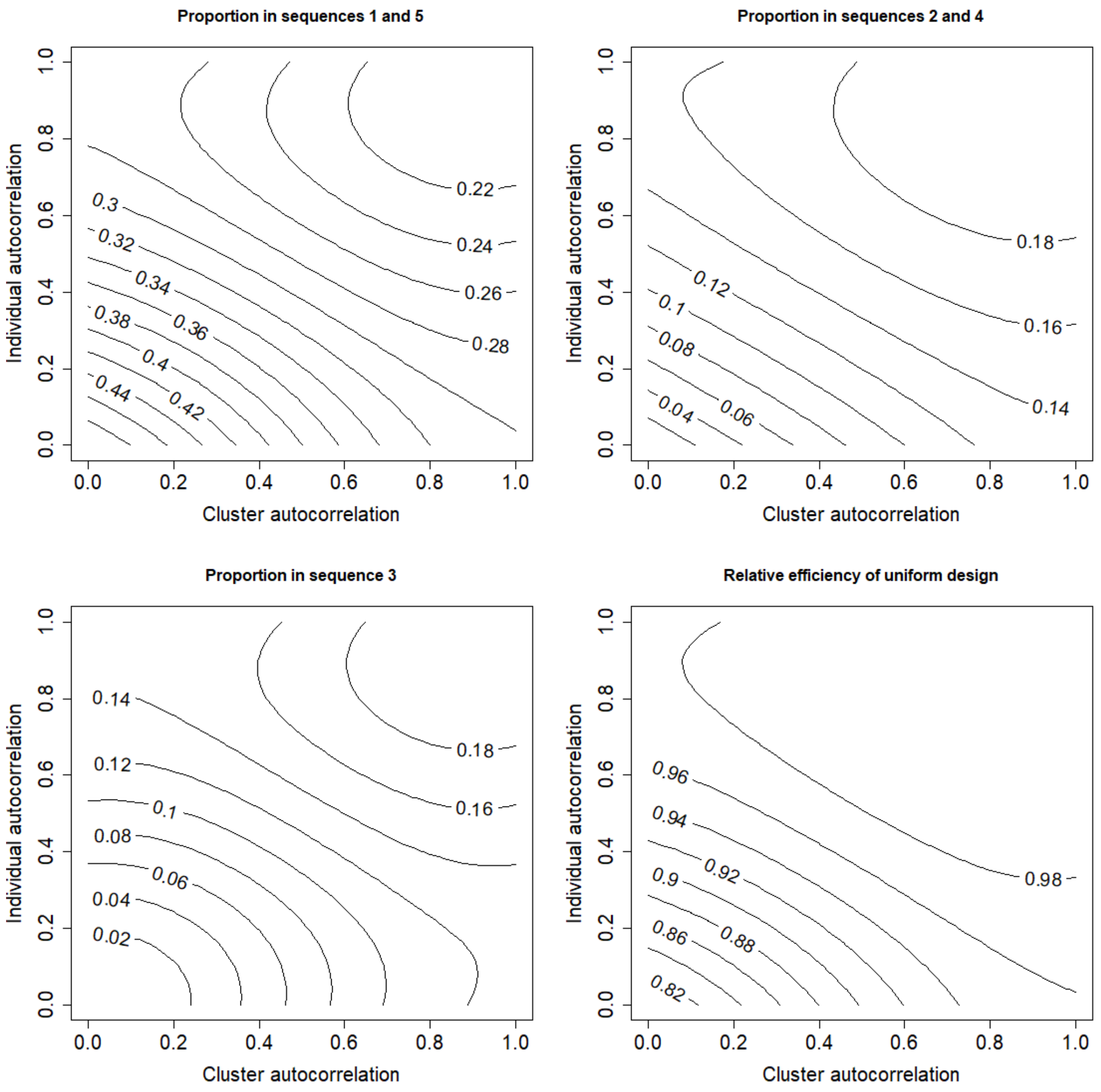

Number of sequences $S = 5$

Intraclass correlation $\rho = 0.05$

Number of subjects per cluster-period $m = 25$

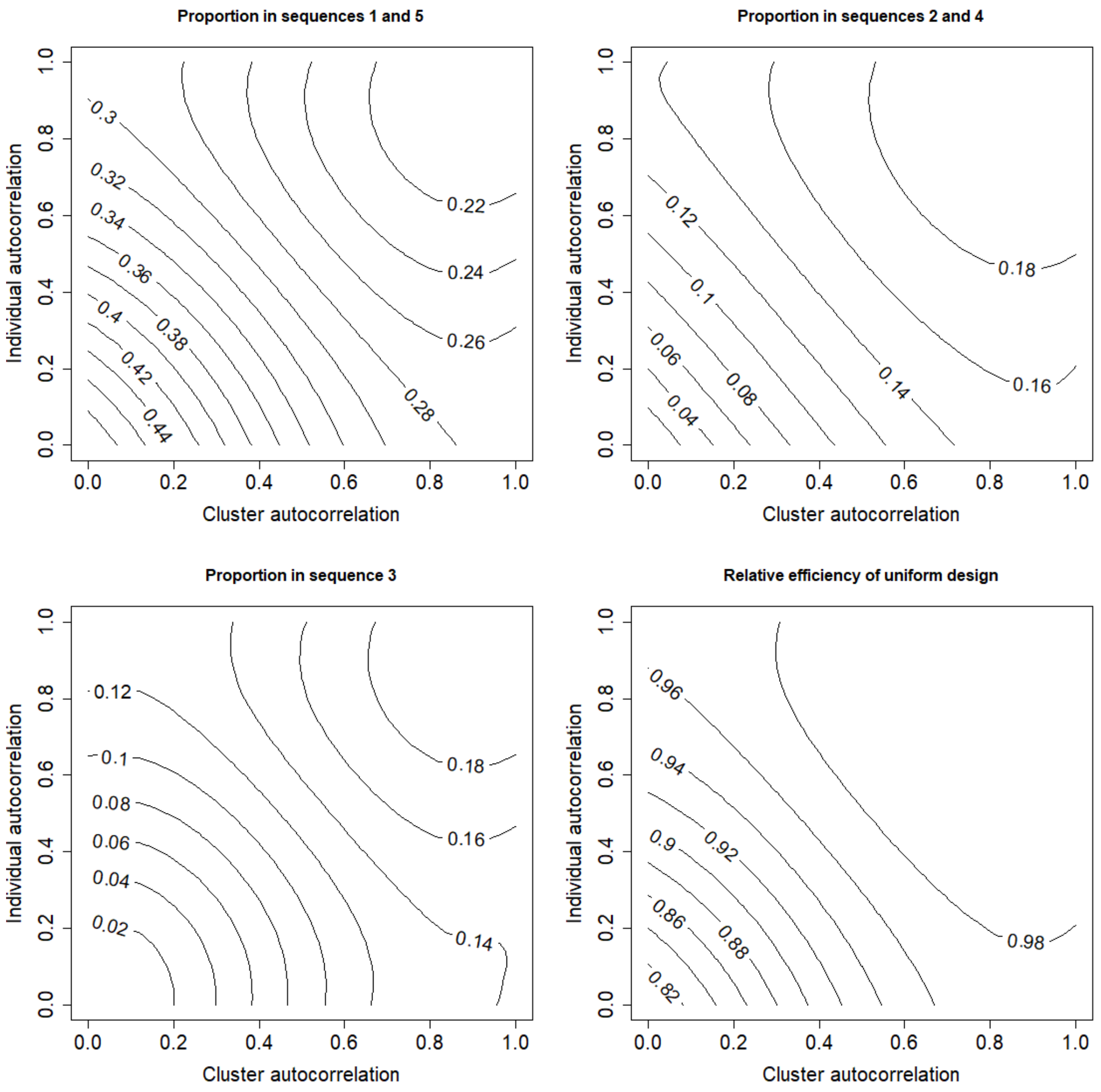

Number of sequences $S = 5$

Intraclass correlation $\rho = 0.0125$

Number of subjects per cluster-period $m = 50$

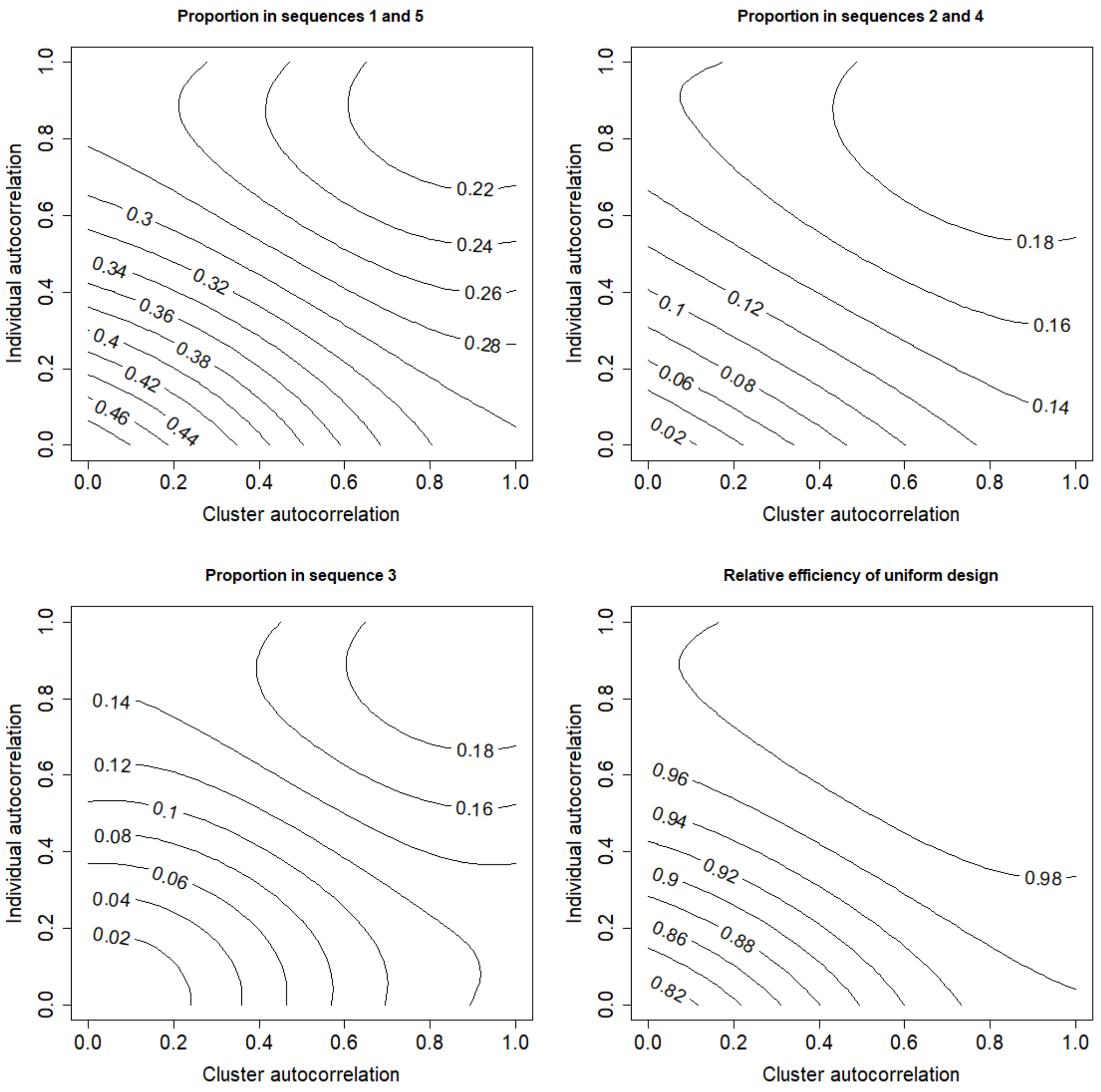

Number of sequences $S = 5$

Intraclass correlation $\rho = 0.025$

Number of subjects per cluster-period $m = 50$

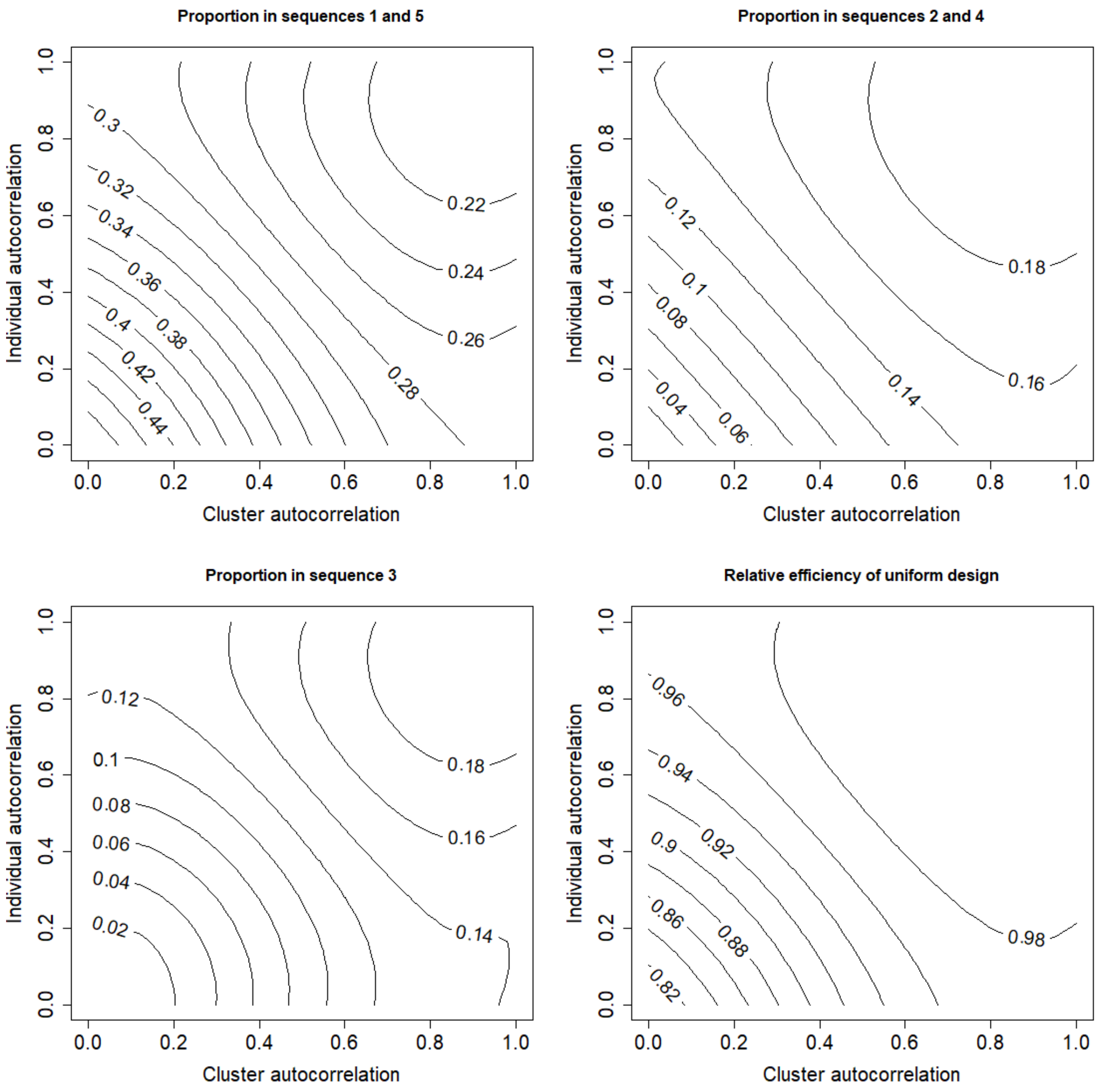

Number of sequences $S = 5$

Intraclass correlation $\rho = 0.05$

Number of subjects per cluster-period $m = 50$

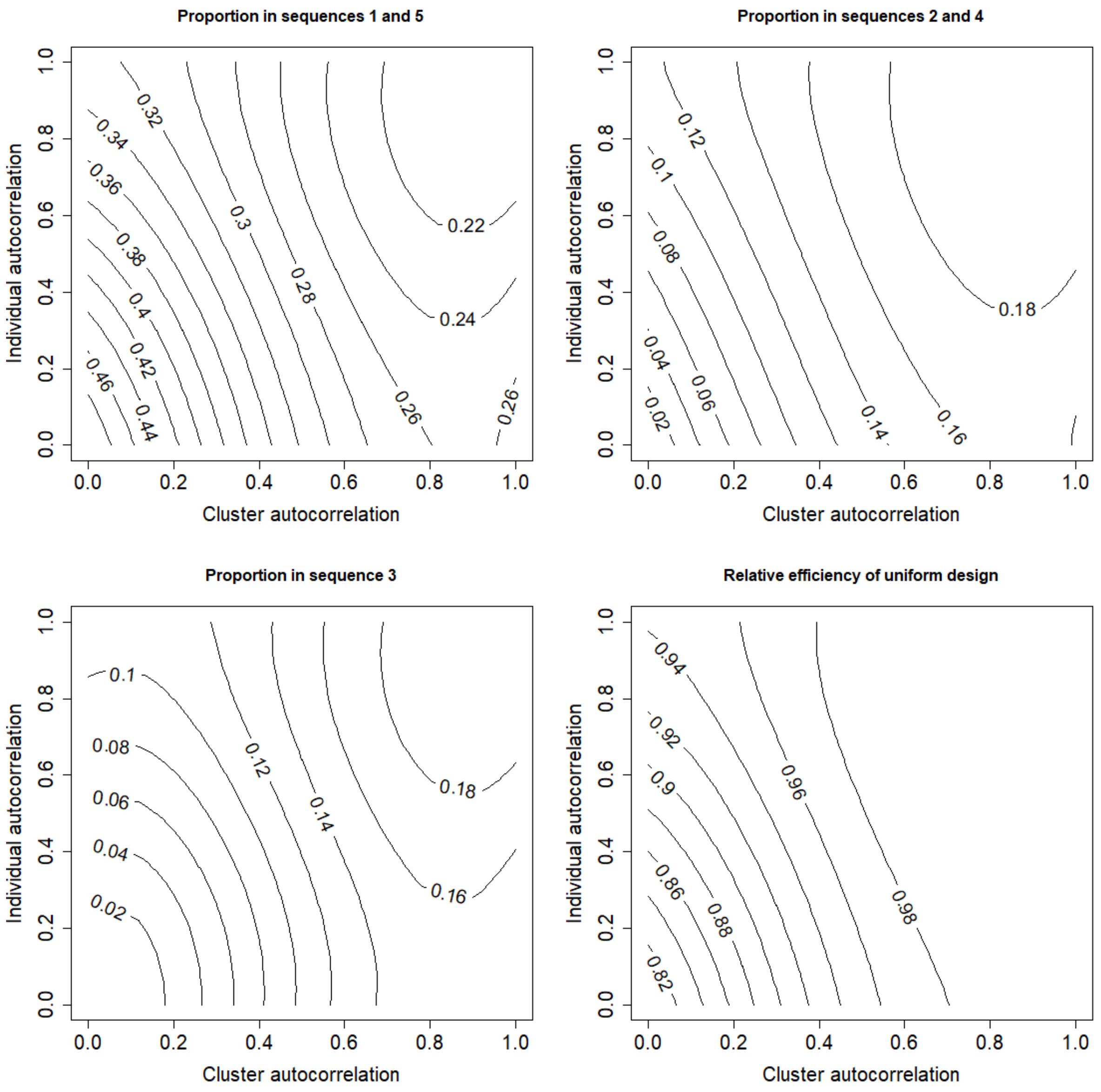

Number of sequences $S = 6$

Intraclass correlation $\rho = 0.0125$

Number of subjects per cluster-period $m = 5$

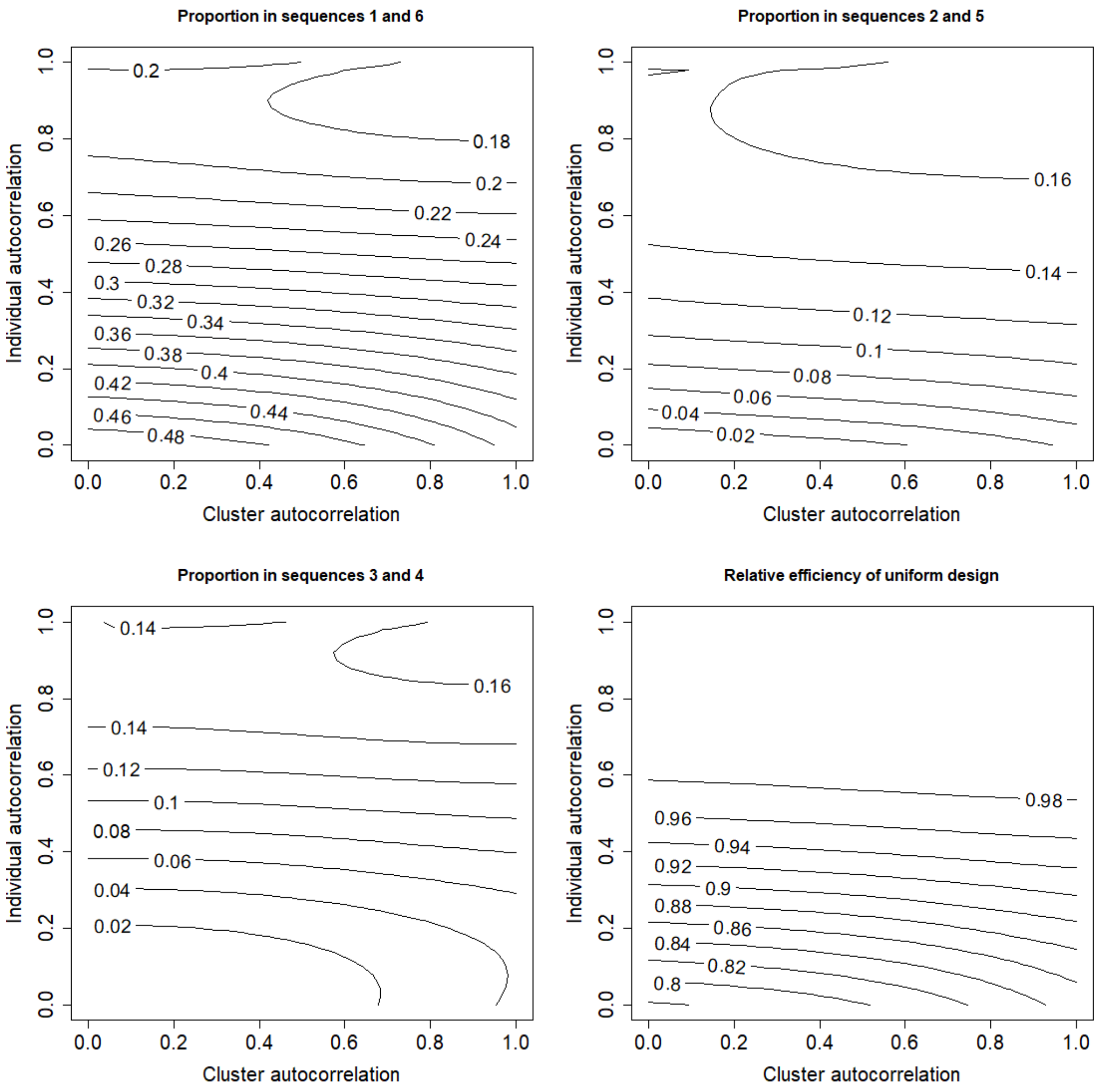

Number of sequences $S = 6$

Intraclass correlation $\rho = 0.025$

Number of subjects per cluster-period $m = 5$

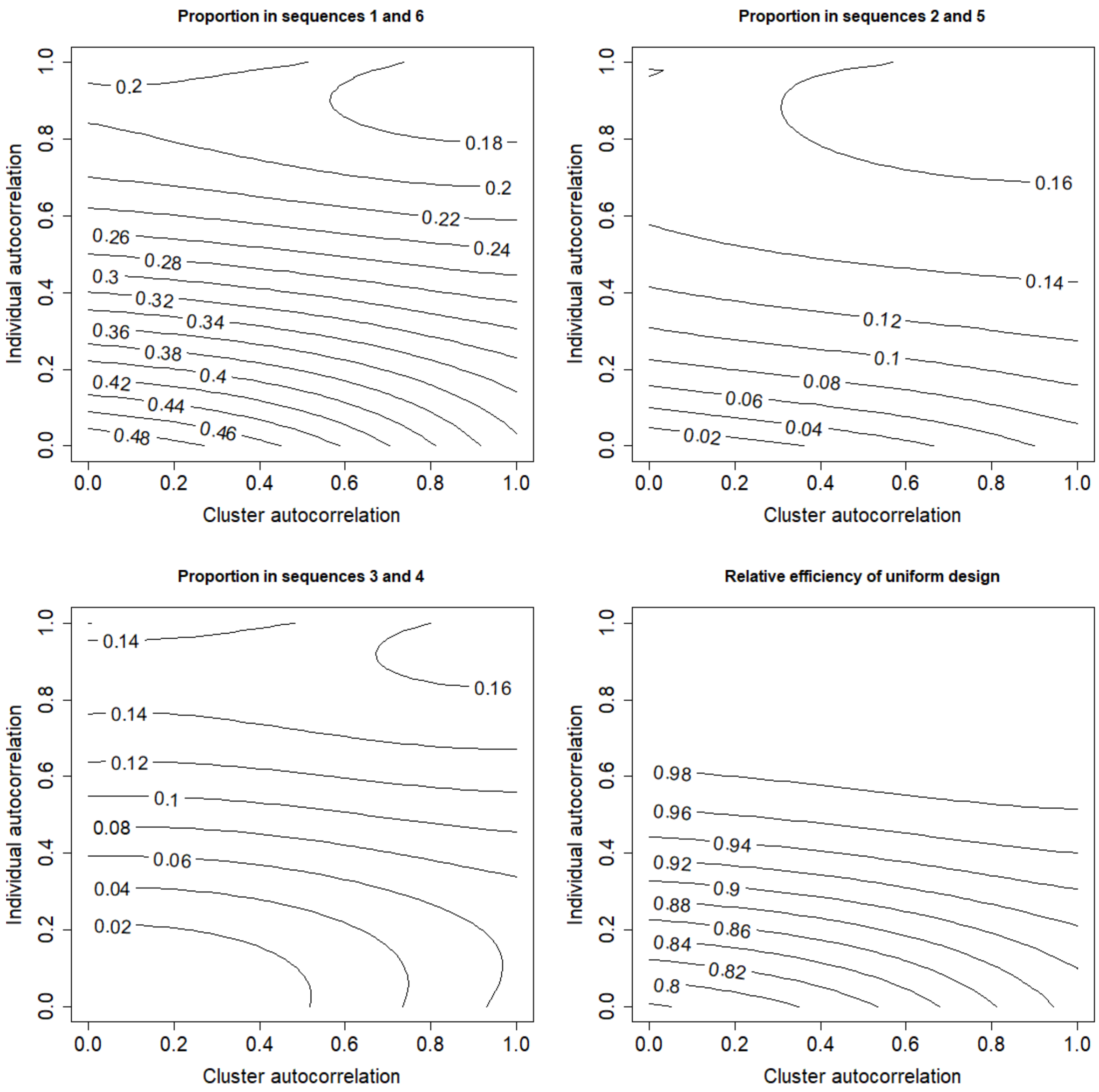

Number of sequences $S = 6$

Intraclass correlation $\rho = 0.05$

Number of subjects per cluster-period $m = 5$

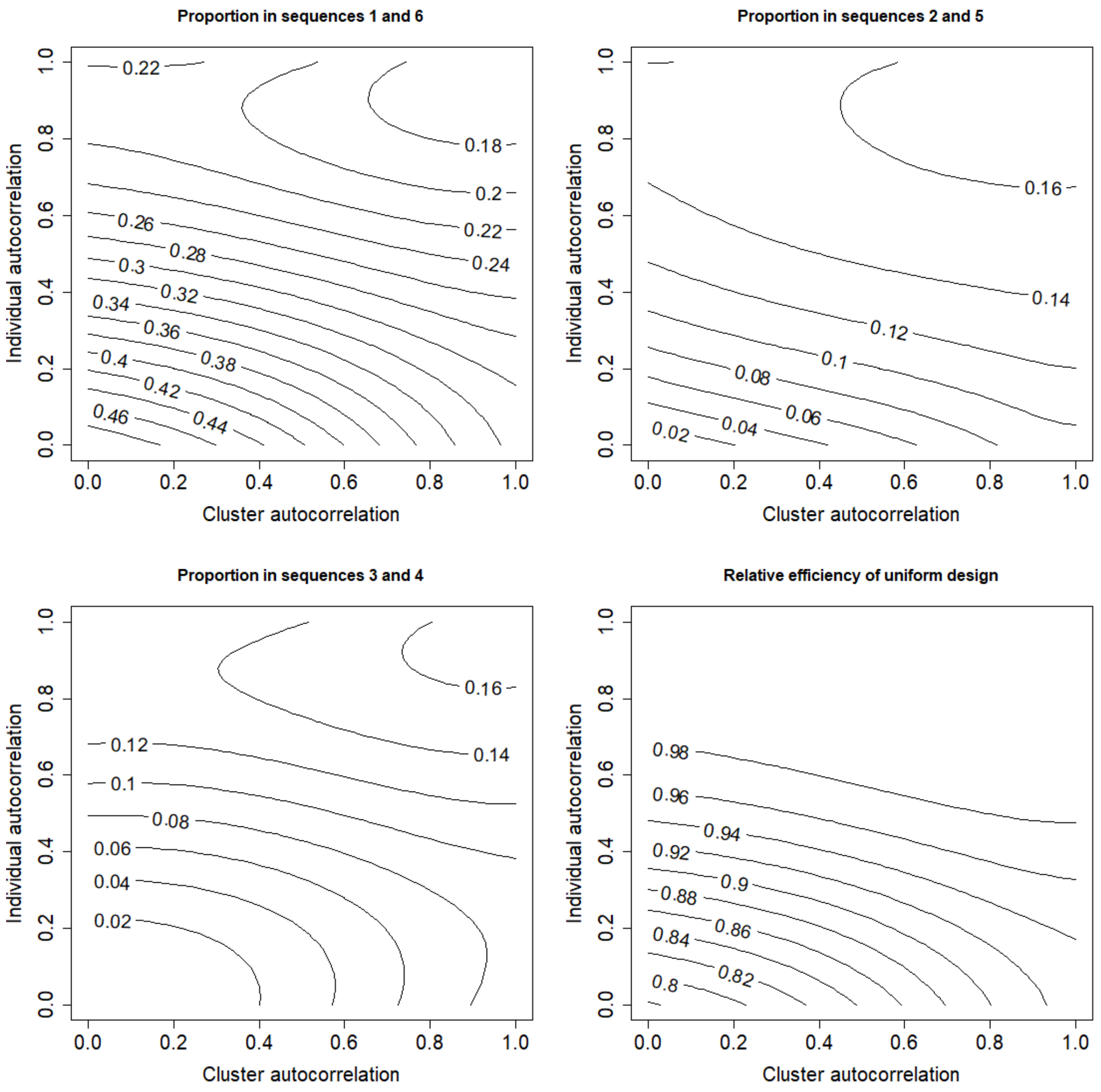

Number of sequences $S = 6$

Intraclass correlation $\rho = 0.0125$

Number of subjects per cluster-period $m = 25$

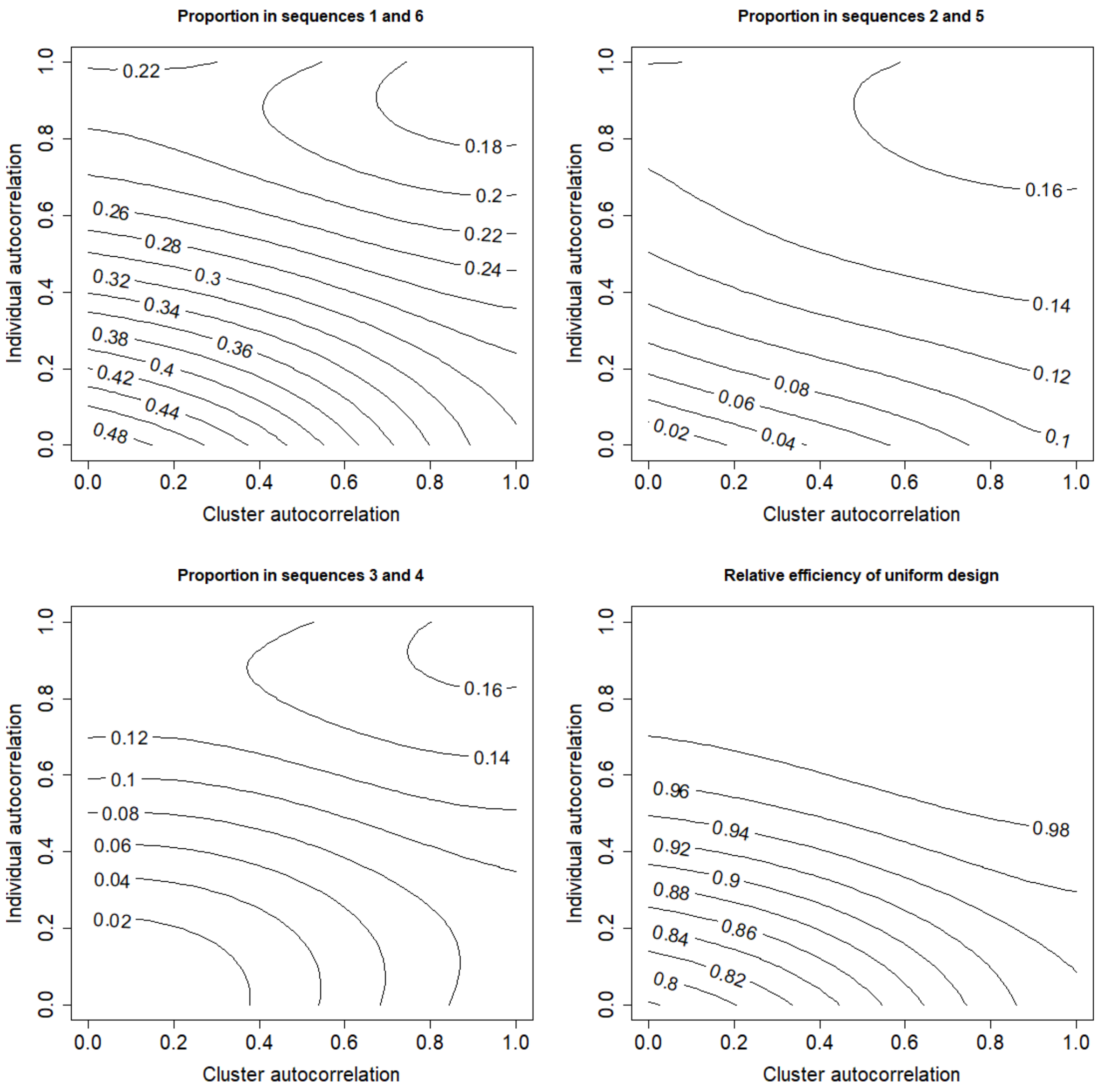

Number of sequences $S = 6$

Intraclass correlation $\rho = 0.025$

Number of subjects per cluster-period $m = 25$

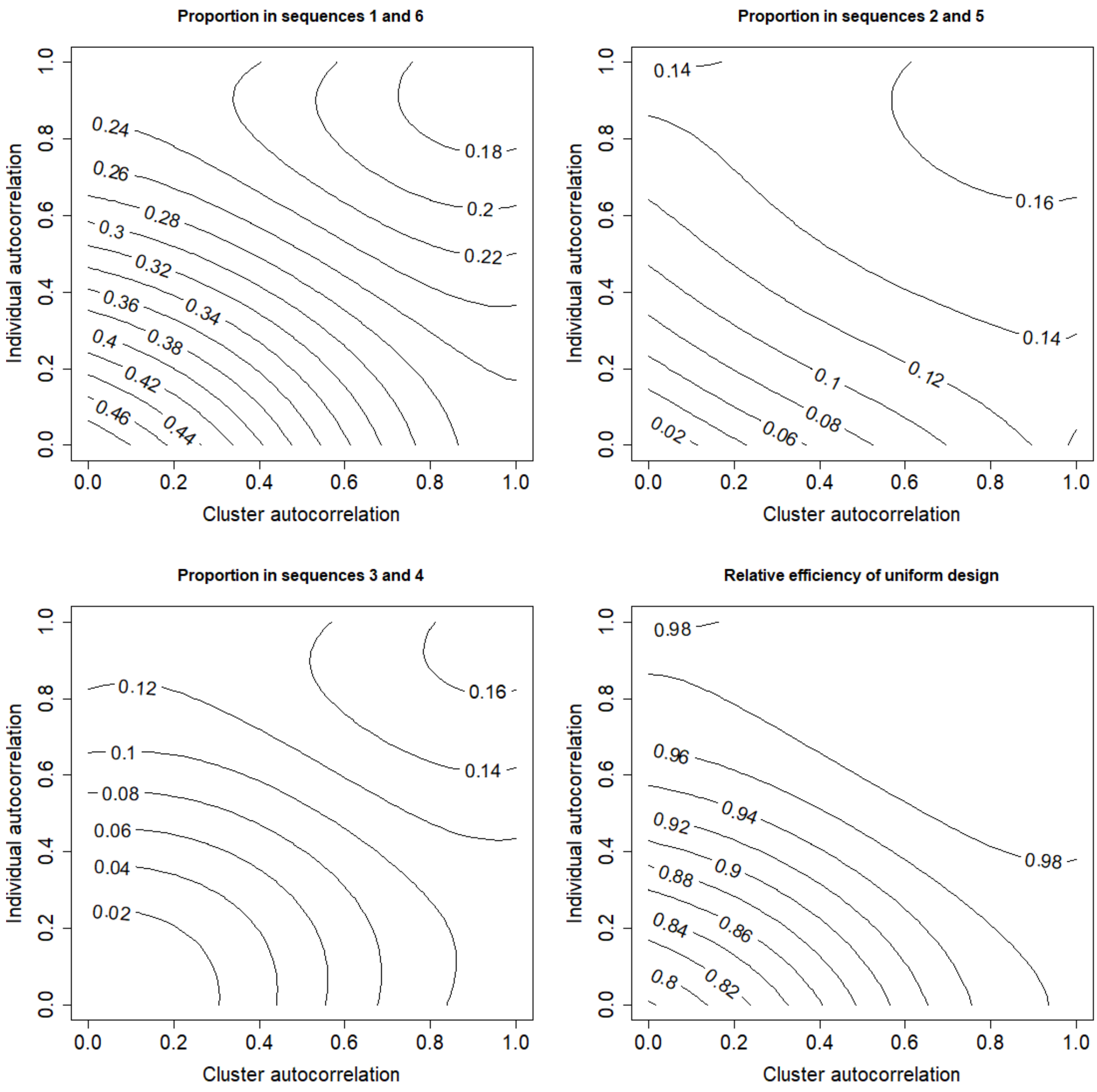

Number of sequences $S = 6$

Intraclass correlation $\rho = 0.05$

Number of subjects per cluster-period $m = 25$

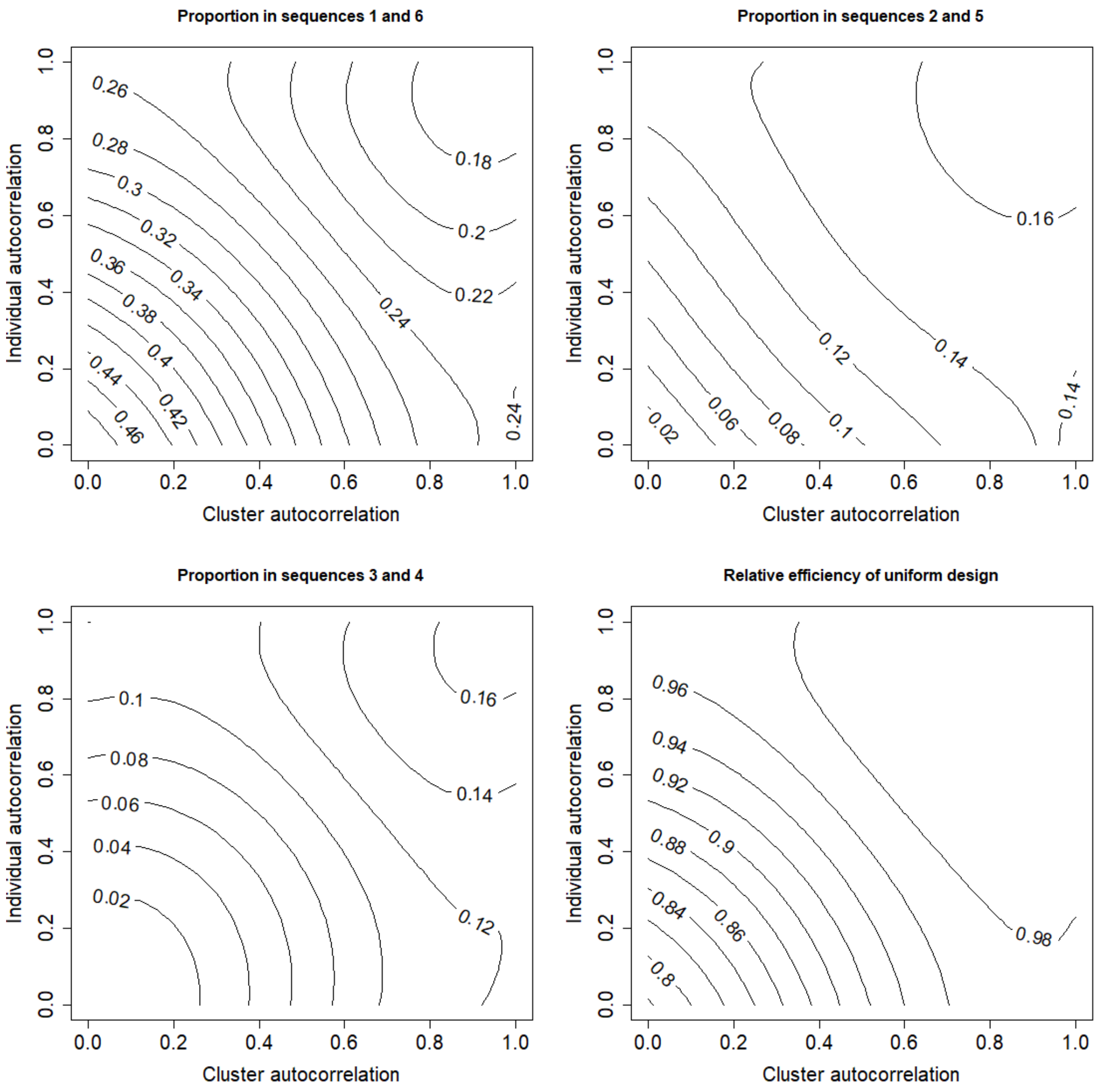

Number of sequences $S = 6$

Intraclass correlation $\rho = 0.0125$

Number of subjects per cluster-period $m = 50$

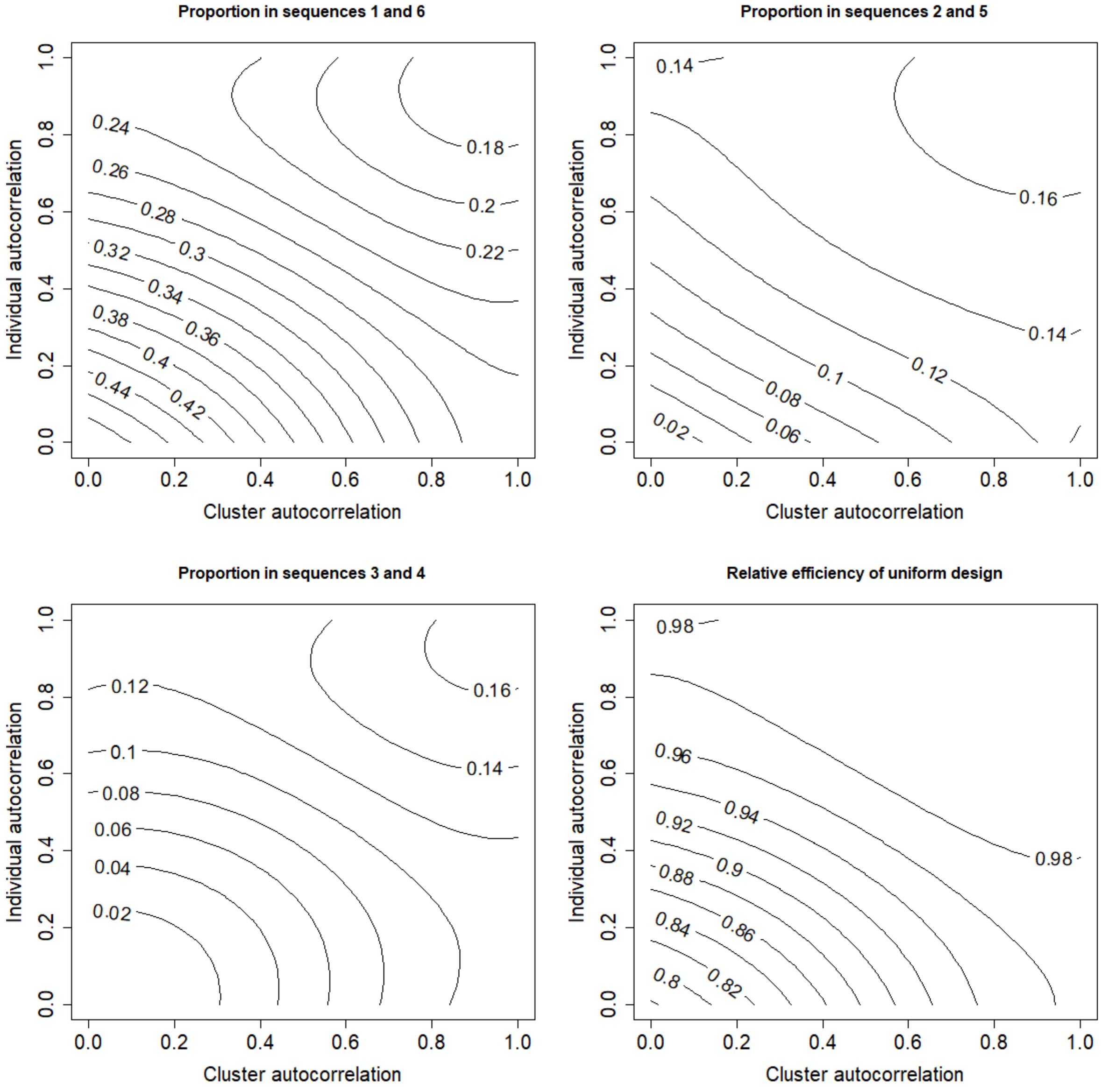

Number of sequences $S = 6$

Intraclass correlation $\rho = 0.025$

Number of subjects per cluster-period $m = 50$

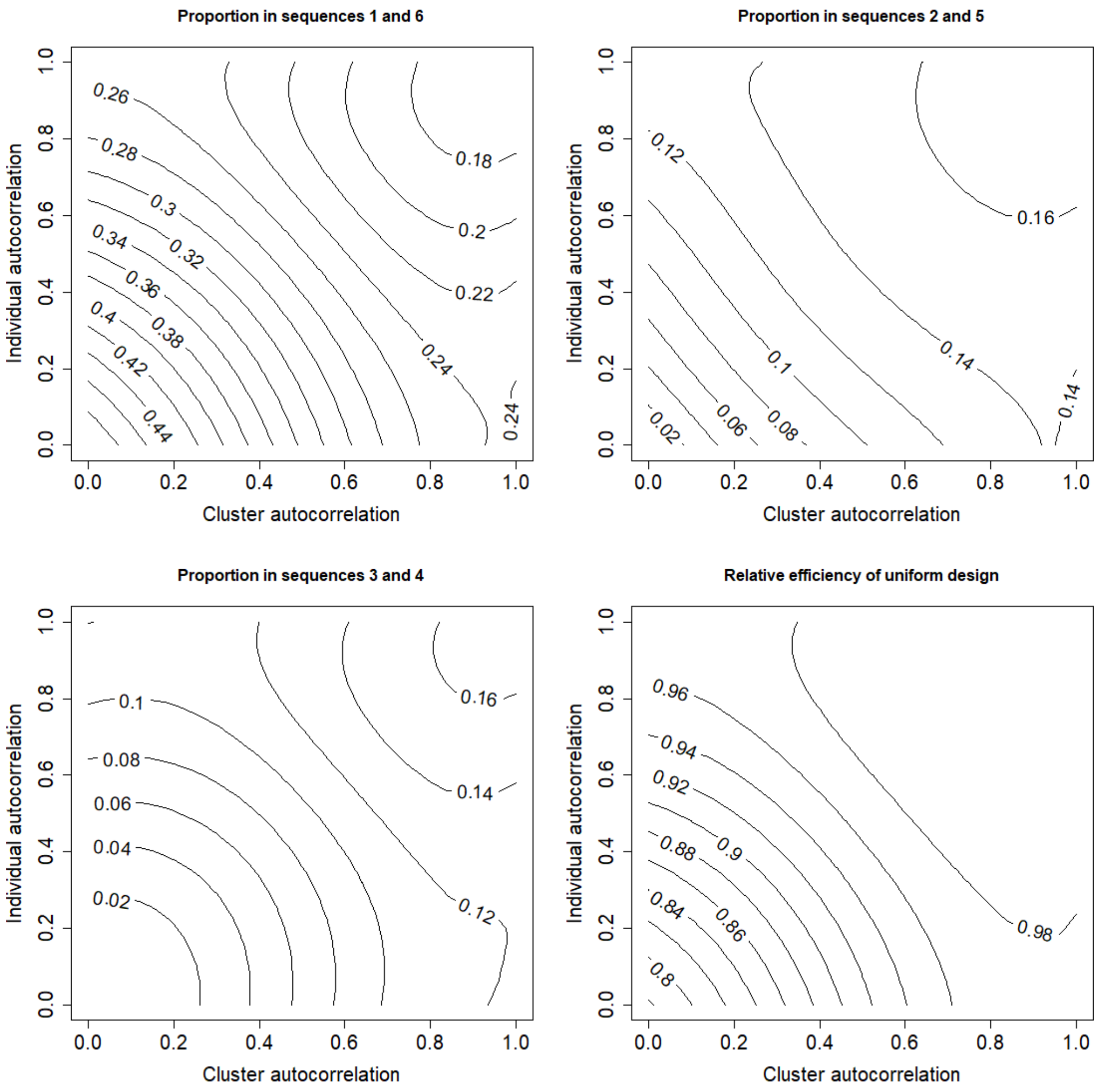

Number of sequences $S = 6$

Intraclass correlation $\rho = 0.05$

Number of subjects per cluster-period $m = 50$

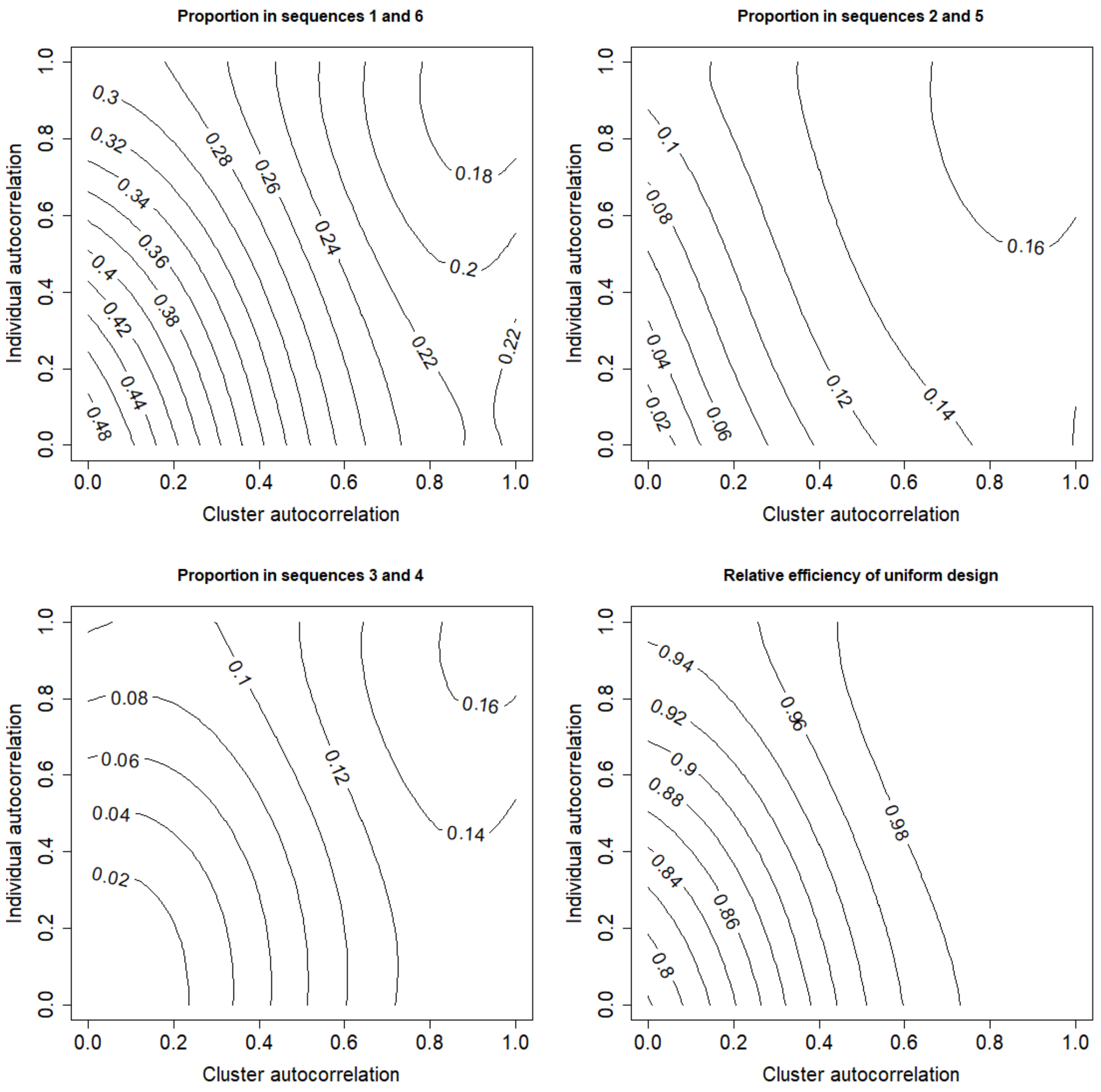

Supplement: S2 Appendix — (PDF) [file pone.0289275.s002.pdf]
