# Supplementary material for: Optimal allocation of clusters in stepped wedge designs with a decaying correlation structure
Source: PLoS One. 2023 Aug 16;18(8):e0289275. doi: 10.1371/journal.pone.0289275 (PMC10431648; doi:10.1371/journal.pone.0289275)
Supplement: S3 Appendix — (PDF) [file pone.0289275.s003.pdf]

Table 11.1: Overview of literature that presents ICC estimates

Source	research field	subjects	clusters
Adams et al. (2004)	primary care	various	primary care practices and practitioners
Agarwal, Awasthi, and Walter (2005)	vitamin A intake	children	administrative blocks and villages
Amorim, Bangdiwala, McMurray, Creighton, and Harrell (2007)	physiology	children and adolescents	public schools
Baskerville, Hogg, and Lemelin (2000)	prevention in primary care	patients	primary care practices
Brandon, Harrison, and Lawton (2012)	education	students	schools
M. K. Campbell et al. (2001)	implementation	patients	primary and secondary care practices
M. K. Campbell, Fayers, and Grimshaw (2005)	implementation	patients	primary and secondary care practices
Carlin and Hocking (1999)	traffic, health	children and adolescents	schools
Elley, Kerse, Chondros, and Robinson (2005)	primary and residential health care	middle-aged and older adults	primary care practices and residential care homes
Feder, Griffiths, Eldridge, and Spence (1999)	secondary prevention of coronary heart disease	patients with acute coronary events	general practices
Feng et al. (1999)	various	various	various
Haddad et al. (2012)	perinatal care	pregnant women	hospitals
Gulliford, Ukoumunne, and Chinn (1999)	health care	adults	postal codes and households

Excerpt from:
Moerbeek, M. & Teerenstra, S. (2016). Power analysis of trials with multilevel data. Boca Raton: CRC Press.
Credits are given to the publisher.

Table 11.1 (continued)

Source	research field	subjects	clusters
Hannan, Murray, Jacobs, and McGovern Jr. (1994)	heart health	individuals	neighborhoods and cities
Hawkins, Van Horn, and Arthur (2004)	substance use	public school students	small to moderate sized towns
Hedberg and Hedges (2014)	education	pupils	schools and districts
Hedges and Hedberg (2007)	education	children, adolescents	schools
Hedges and Hedberg (2014)	education	students	schools
Hutchison (2009)	education	pupils	primary and secondary schools
Ip, Wasserman, and Barkin (2011)	pediatrics	parents	pediatric practices
Jacob, Zhu, and Bloom (2010)	education	students	classes and schools
Janega et al. (2004a)	nutrition intervention	students	schools
Janega et al. (2004b)	alcohol, tobacco, drugs	students	middle schools
Janjua, Khan, and Clemens (2006)	injection practices in developing countries	children, adults	groups within geographic boundaries
Kelcey and Phelps (2013)	education	teachers	schools
Kelcey and Phelps (2014)	education	teachers	schools
Knox and Chondros (2004)	primary care	patients	general practitioners
Konstantopoulos (2009)	education	students	classes and schools
Littenberg and MacLean (2006)	diabetes in primary care	adults with diabetes	primary care practices
Lajos et al. (2014)	perinatal care	births	referral hospitals
Martinson, Murray, Jeffery, and Hennrikus (1999)	worksite health promotion	employees	businesses

Excerpt from:
Moerbeek, M. & Teerenstra, S. (2016). Power analysis of trials with multilevel data. Boca Raton: CRC Press.
Credits are given to the publisher.

Table 11.1 (continued)

Source	research field	subjects	clusters
Metcalf, Scragg, Stewart, and Scott (2007)	nutrition	children	neighboring houses
Murray and Hannan (1990)	tobacco and drug use	students	junior and senior high schools
Murray et al. (1994)	adolescent smoking	students	schools
Murray and Short (1995)	alcohol use	young adults	communities
Murray and Short (1996)	alcohol use	pupils	schools
Murray and Short (1997)	tobacco use	adolescents	schools
Murray, Phillips, Birnbaum, and Lytle (2001)	nutrition	students	schools
Murray et al. (2002)	tobacco use	adolescents	classrooms
Murray, Van Horn, et al. (2006)	alcohol, tobacco and drug use	adolescents	communities
Murray, Stevens, et al. (2006)	physical activity	sixth grade girls	schools
Murray, Blitstein, Hannan, Baker, and Lytle (2007)	eating and nutrition	7th and 8th graders	middle schools
Pagel et al. (2011)	perinatal care	mothers and newborns	communities in developing countries
S. L. Pals, Beaty, Posner, and Bull (2009)	HIV/STD prevention	young African American and Hispanic females	neighborhoods
Parker, Evangelou, and Eaton (2005)	cholesterol education and research	patients	primary care practices
Piaggio et al. (2001)	antenatal care	pregnant women	clinics
Preisser, Reboussin, Song, and Wolfson (2007)	underage drinking	adolescents	cities and counties
Reading, Harvey, and McLean (2000)	maternal and child health	families with infants under 1 year	practices

Excerpt from:
Moerbeek, M. & Teerenstra, S. (2016). Power analysis of trials with multilevel data. Boca Raton: CRC Press.
Credits are given to the publisher.

Table 11.1 (continued)

Source	research field	subjects	clusters
Roudsari, Nathens, Koepsell, Mock, and Rivara (2006)	trauma	patients	trauma centers
Roudsari, Fowler, and Nathens (2007)	childhood trauma	children	trauma centers
Resnocow et al. (2010)	smoking	pupils	high schools
Rowe, Lama, Onikpo, and Deming (2002)	health care	patients	health facility
Scheier, Griffin, Doyle, and Botvin (2002)	drug abuse	students	schools
Schochet (2008)	education	students	schools
Siddiqui et al. (1996)	smoking	students	classes and schools
Slymen and Hovell (1997)	tobacco and alcohol use	adolescents	orthodontists
Slymen et al. (2003)	tobacco and alcohol use	migrant adolescents	schools
Smeeth and Ng (2002)	assessment and management of older people	adults 75 years and older	general practices
Taljaard et al. (2008)	maternal and perinatal health	mothers and newborns	hospitals
D. M. Thompson, Fernald, and Mold (2012)	health care	patients	general practices
Westine, Spybrook, and Taylor (2014)	education	students	schools
Xu and Nichols (2010)	education	students	classes and schools
Yelland, Salter, Ryan, and Laurence (2011)	pathology testing	patients	primary care practices
Zhu, Jacob, Bloom, and Xu (2012)	education	students	classes and schools

Excerpt from:
Moerbeek, M. & Teerenstra, S. (2016). Power analysis of trials with multilevel data. Boca Raton: CRC Press.
Credits are given to the publisher.
